# Dual-Wavelength Gated *oxo*-Diels–Alder Photoligation

**DOI:** 10.1021/acs.orglett.1c00015

**Published:** 2021-02-23

**Authors:** Marc Villabona, Sandra Wiedbrauk, Florian Feist, Gonzalo Guirado, Jordi Hernando, Christopher Barner-Kowollik

**Affiliations:** † Department de Química, Universitat Autònoma de Barcelona, Edifici C/n, Campus UAB, 08193 Cerdanyola del Vallès, Spain; ‡ Centre for Materials Science, School of Chemistry and Physics, Queensland University of Australia (QUT), 2 George Street, Brisbane, Queensland 4000, Australia

## Abstract

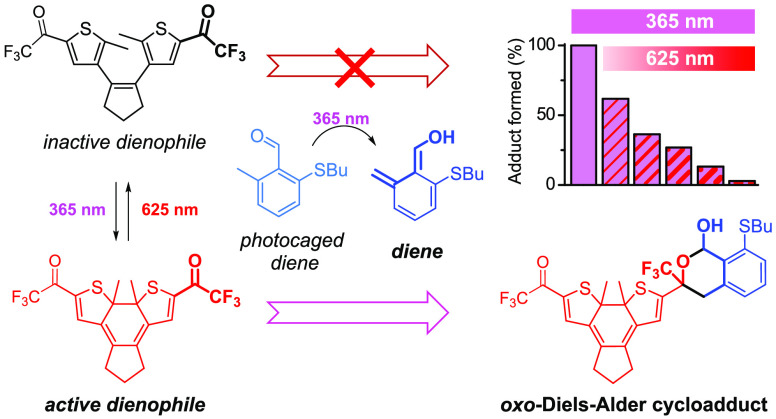

The control of chemical functionalization with orthogonal light stimuli paves the way toward manipulating materials with unprecedented spatiotemporal resolution. To reach this goal, we herein introduce a photochemical reaction system that enables two-color control of covalent ligation via an *oxo*-Diels–Alder cycloaddition between two separate light-responsive molecular entities: a UV-activated photocaged diene based on *ortho*-quinodimethanes and a carbonyl dienophile appended to a diarylethene photoswitch, whose reactivity can be modulated upon illumination with UV and visible light.

By enabling chemical functionalization of molecules and materials with high efficiency and spatiotemporal control, photoinduced ligation reactions^1^ are finding applications in a variety of fields ranging from polymer network formation^2^ to surface patterning,^3^ 3D printing,^4^ and bioconjugation.^5^ In most of the cases, these photoreactions are driven by irradiation with a single light source and, therefore, their time and spatial profile evolution is determined by illumination intensity and the reagents’ concentration and properties. A much more precise control of photoinduced reactivity can, however, be achieved by regulating the ligation processes with two independent optical signals, i.e., two different colors of light that serve as “and” gates for the λ-orthogonal activation and deactivation of the desired reaction on demand.^1b,1c^ Such a strategy can, among other advantages, grant access to novel lithographic processes^6^ and finely controlled postmodification strategies for materials,^7^ which constitute two key concepts for the development of advanced functional structures.^8^

Current methodologies applied to the dual-wavelength control of photoligation chemistry involve time or spatially resolved two-color irradiation of one of the reagents of a bimolecular reaction, which can be either molecules capable of undergoing photocycloaddition and photocycloreversion processes (e.g., anthracenes^7c^) or molecular photoswitches whose reactivity can be reversibly toggled upon photoisomerization.^6b−6e,7a,7b,9^ To control light-induced reactivity with finely selected colors of light, we herein pioneer a reaction strategy that ceases photochemical bond formation upon the simultaneous irradiation of two photoreactive entities (Figure 1a): a photocaged reagent, which becomes activated upon irradiation with one color of light (λ_1_), and a photoswitch reversibly isomerizing between reactive and nonreactive states under illumination with two different wavelengths (λ_1_ and λ_2_). As a result, bond formation proceeds at λ_1_ and is halted upon the dual impact of λ_1_ and λ_2_.

**Figure 1:**
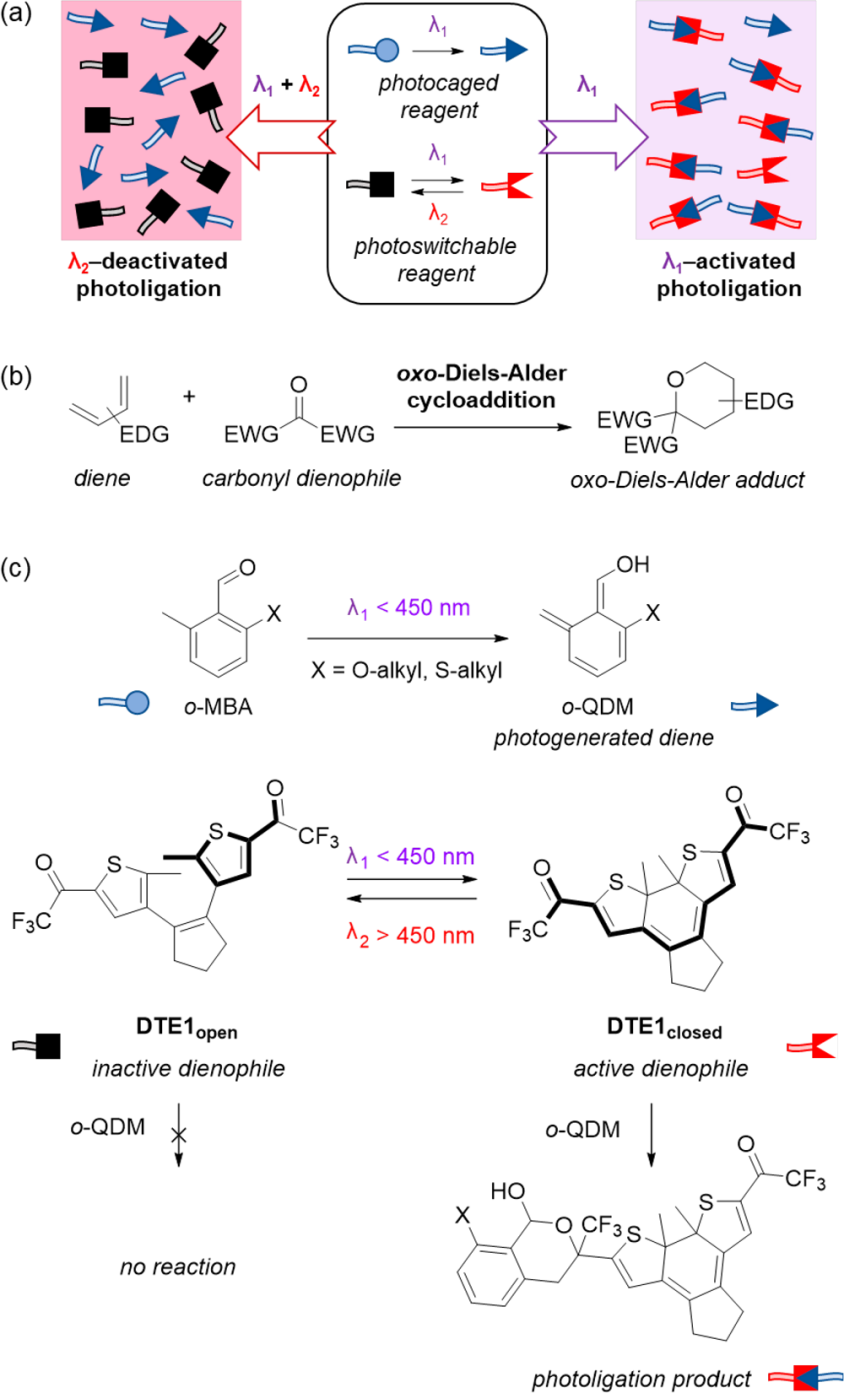
(a) Strategy for dual-wavelength control of photoligation reactions by combining photocaged and photoswitchable reagents. (b) Normal electron demand *oxo*-Diels–Alder cycloaddition selected as a photoligation reaction (EDG = electron-donating group; EWG = electron-withdrawing group). (c) General structures of the photogenerated *o*-quinodimethane dienes and the photoswitchable DAE-trifluoromethyl ketone dienophile employed, which should undergo *oxo*-Diels–Alder cycloaddition selectively for the closed state of **DTE1**.

To reach this goal, we herein focus our attention on a photoligation process that has been seldomly explored for light-induced chemical functionalization: the *oxo*-Diels–Alder cycloaddition, a *hetero*-Diels–Alder reaction between dienes and carbonyl groups (Figure 1b).^10^ When driven under normal electron demand, catalyst-free *oxo*-Diels–Alder cycloadditions are highly favored for electron-rich dienes and electron-poor dienophiles,^11^ a condition on which we capitalize herein to reach two-color control of photoreactivity at ambient temperature. Specifically, we select *o*-quinodimethanes (*o*-QDMs) photochemically generated from *o*-methyl benzaldehydes (*o*-MBAs) as electron-rich dienes (Figure 1c),^10,12^ which have been widely exploited for other types of photoligation reactions (e.g., *homo*-Diels–Alder cycloadditions^13^ and self-dimerization^14^). As for the electron-poor dienophile, we choose trifluoromethyl ketones,^15^ which are tethered to a diaryl-ethene photoswitch (DAE) to reversibly alter their reactivities upon photoisomerization (Figure 1c). DAEs^16^ have already been used to photomodulate *homo*-Diels–Alder cycloadditions by generating and removing reactive diene functionalities when interconverting between their open and closed states;^17^ however, it is another property of these systems that we take advantage of: the change in electronic communication between the substituents of the aryl rings upon photoisomerization.^7a,16^ For the herein utilized symmetric dithyenylethene **DTE1**, the carbonyl group electron density of the two electron-withdrawing trifluoromethyl carbonyl side groups should be dramatically decreased in the closed state of the switch, where they are electronically coupled (Figure 1c). Thus, these interdependent entities become more active dienophiles for the photogenerated *o*-QDM dienes, thereby allowing for photomodulation of the corresponding *oxo*-Diels–Alder reaction.

To synthesize **DTE1**, a classic approach for the preparation of trifluoromethyl ketones was employed, which consists of the trifluoroacetylation of organometallic reagents.^18^ Thus, when **1**,^19^ a common intermediate for the preparation of a variety of DAE switches, was sequentially treated with *tert*-butyllithium and ethyl trifluoroacetate in anhydrous THF, the open isomer of **DTE1** (**DTE1**_**open**_) was readily obtained in 60% yield (Figure 2a). **DTE1** preserves the characteristic photochromic behavior of DAEs. First, its colorless open isomer (λ_abs_ < 400 nm, Figure 2b) rapidly transforms into the closed **DTE1**_**closed**_ state upon irradiation with UV (or even violet) light (Figure S1). In our hands, this light-induced process proceeded with a high quantum yield (Φ_o-c_ = 0.37 in toluene) and efficiency, as we determined by ^1^H NMR that the photostationary state produced at a λ_exc_ of 365 nm in toluene contained up to 96% of the closed isomer. **DTE1**_**closed**_ was found to be thermally stable in the dark at room temperature, both in solution and in the solid state. As shown in Figure 2b, it strongly absorbs in the visible region (λ_abs,max_ = 635 nm in toluene) and, when irradiated with green or red light, quantitatively back-photoisomerizes to the open isomer with a modest quantum yield (Φ_c-o_ = 0.031 in toluene, Figure S1). Finally, **DTE1** shows excellent fatigue resistance and can be reversibly interconverted between its open and closed states without apparent photodegradation (Figure 2c).

**Figure 2:**
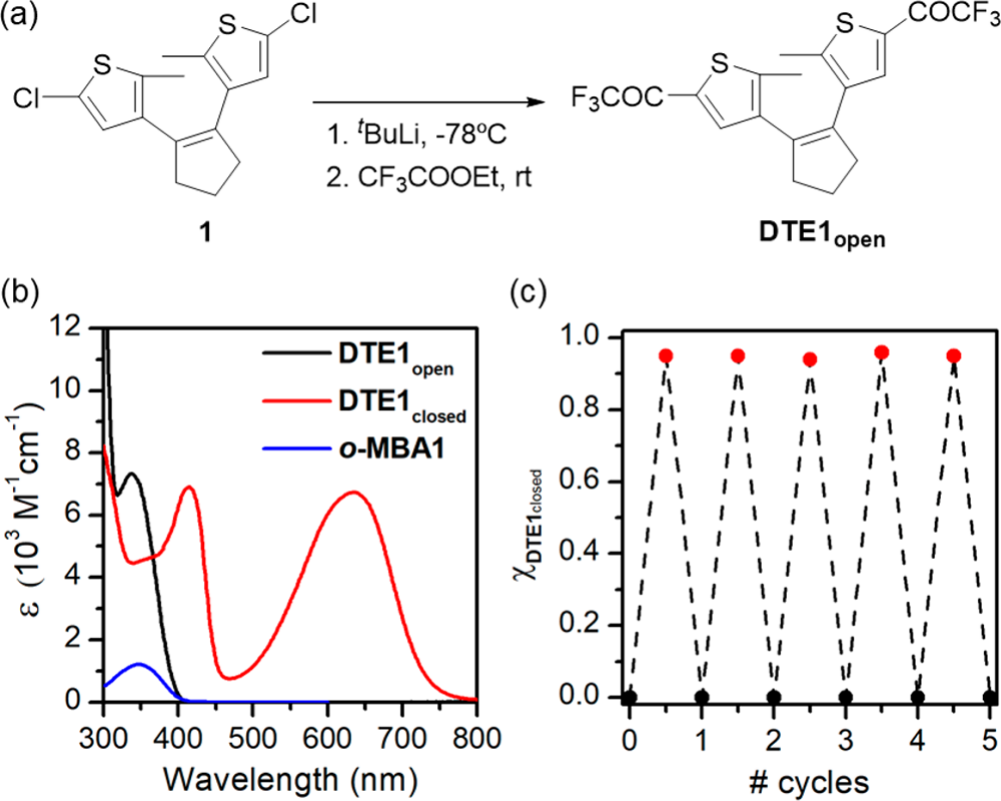
(a) Synthesis of **DTE1**_**open**_. (b) UV–vis absorption spectra of **DTE1**_**open**_, **DTE1**_**closed**_, and ***o*****-MBA1** (X = SBu, Figure 3a) in toluene. (c) Variation of the molar fraction of **DTE1**_**closed**_ (as determined from UV–vis absorption data) upon consecutive cycles of irradiation of a toluene solution of this compound with UV (λ_exc_ = 365 nm) and visible (λ_exc_ = 650 nm) light to trigger photoisomerization between its open and closed states.

According to our molecular design, the trifluoromethyl carbonyl groups in **DTE1**_**closed**_ should efficiently undergo *oxo*-Diels–Alder cycloaddition with electron-rich dienes. To validate this hypothesis, we explored the reaction between **DTE1**_**closed**_ and methylbenzaldehyde *o***-MBA1** (Figure 3a), a model photocaged precursor of diene ***o***-**QDM1** that was prepared following the methodology previously reported by us (Scheme S1).^20^***o***-**MBA1** was selected on the basis of the well-documented capacity of *o*-MBA derivatives to undergo sequential intramolecular H-transfer and isomerization upon photoexcitation, which produces *o*-QDM dienes that can be trapped in a Diels–Alder reaction with electron-deficient enes (Figure 1c).^6b,20,21^ In addition, by introducing an alkylthio group in ***o*****-MBA1**, its absorption spectrum bathochromically shifts relative to regular nonsulfurated *o*-MBAs, reaching maximal photoreactivity at λ_exc_ ∼ 350–390 nm (Figure 2b).^13,20^ At such spectral range, **DTE1**_**open**_ also shows high photoconversion into **DTE1**_**closed**_, thus ensuring efficient light activation of both substrates of the cycloaddition reaction using a single irradiation wavelength.

**Figure 3:**
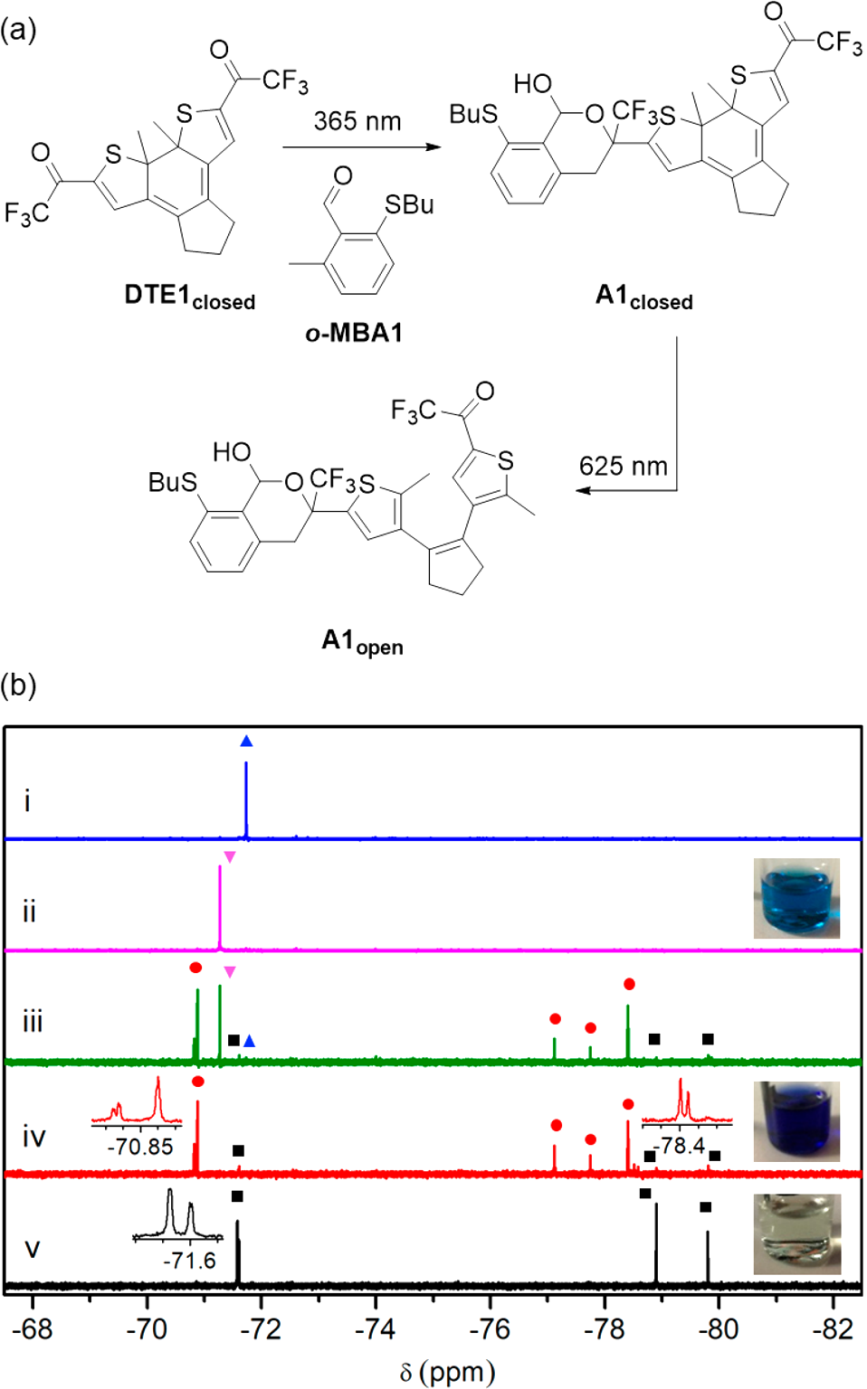
(a) *oxo*-Diels–Alder reaction of **DTE1**_**closed**_ with the diene photogenerated from ***o*****-MBA1** under UV illumination (LED λ_max_ = 365 nm, Figure S2) and ambient temperature. The adduct **A1**_**closed**_ can be converted to its open isomer **A1**_**open**_ upon irradiation with visible light (LED λ_max_ = 625 nm, Figure S2). (b) ^19^F NMR spectra (565 MHz, CD_3_CN) of (i) **DTE1**_**open**_; (ii) **DTE1**_**closed**_; (iii) a mixture of **DTE1**_**closed**_ and ***o*****-MBA1** after UV illumination (LED λ_max_ = 365 nm, 0.017 mW cm^–2^) for 45 min in deuterated acetonitrile; (iv) a mixture of **DTE1**_**closed**_ and ***o*****-MBA1** after UV illumination (LED λ_max_ = 365 nm, 0.017 mW cm^–2^) for 90 min in deuterated acetonitrile, which resulted in nearly quantitative transformation into **A1**_**closed**_; (v) **A1**_**closed**_ upon irradiation with visible light (LED λ_max_ = 625 nm, 35 mW cm^–2^) for 2 min in deuterated acetonitrile, which led to quantitative photoisomerization into **A1**_**open**_. For clarity, the ^19^F NMR resonances in each spectrum arising from **DTE1**_**open**_ (blue ▲), **DTE1**_**closed**_ (pink ▼), **A1**_**closed**_ (red ●), and **A1**_**open**_ (black ■) are indicated with different symbols. The changes in color of the reaction mixture before illumination (ii, **DTE1**_**closed**_) and after UV (iv, **A1**_**closed**_) and subsequent visible (v, **A1**_**open**_) irradiation are shown as insets in (b).

The photoligation reaction between the activated dienophile **DTE1**_**closed**_ and ***o*****-MBA1** was tested at ambient temperature in acetonitrile and toluene under UV illumination (LED λ_max_ = 365 nm, Figures 3a and S2), and equivalent results were obtained in both solvents. A large excess of ***o*****-MBA1** was employed (30:1 molar ratio) to favor complete conversion into the cycloaddition products, while minimizing competing UV light absorption by **DTE1**_**closed**_. This, in combination with the photochromic properties of **DTE1**, made light-induced transformation of the closed isomer into the open form negligible at our experimental conditions, as proven by the very low intensity registered along the process for the ^19^F NMR signal of **DTE1**_**open**_ (δ = −71.73 ppm) (Figure 3b). In addition, photodegradation of **DTE1**_**closed**_ was not observed either under equivalent illumination conditions in the absence of ***o*****-MBA1** (Figure S3). Thus, the evolution of new resonances in the ^19^F NMR spectrum of the reaction mixture under UV irradiation selectively reported on the products formed by direct photoligation between **DTE1**_**closed**_ and ***o***-**MBA1**. In practice, a complex set of NMR resonances was obtained after illumination for 90 min (Figure 3b), where (a) the ^19^F NMR resonance of **DTE1**_**closed**_ at δ = −71.26 ppm fully vanished, thus indicating a complete reaction with ***o***-**MBA1**; (b) three slightly downfield shifted signals appeared at δ = −70.81, −70.82, and −70.87 ppm, which could be assigned to the trifluoromethyl carbonyl groups of the photoproducts; (c) four additional intense peaks developed at rather lower chemical shifts (δ = −77.12, −77.74, −78.40, and −78.41 ppm), which is compatible with the CF_3_ substituents of the isochroman moiety formed through cycloaddition.^22^ It must be noted that, though such a diversity of NMR resonances could indicate the occurrence of competitive photoreactions, they could simply originate from the large number of regio- and stereoisomers that can be produced by light-induced *oxo*-Diels–Alder cycloaddition between **DTE1**_**closed**_ and ***o***-**MBA1** (Scheme S2).

Interestingly, the complex ^19^F NMR spectrum obtained for the photoreaction mixture of **DTE1**_**closed**_ and ***o***-**MBA1** was largely simplified after irradiation with visible light (LED λ_max_ = 625 nm, Figure S2), where only two sets of different signals attributable to trifluoromethyl carbonyl (δ = −71.57 and −71.60 ppm) and trifluoromethyl isochromanyl (δ = −78.90 and −79.80 ppm) groups were found (Figure 3b). As color bleaching of the sample was concomitantly observed (Figures 3b and S4), we hypothesize that quantitative ring-opening of the DAE structure of the cycloadducts formed by the reaction between **DTE1**_**closed**_ and ***o***-**MBA1** (**A1**_**closed**_) occurred, thereby yielding the corresponding colorless open-ring products with a lower number of stereoisomers (**A1**_**open**_, Figure 3a and Scheme S2). Actually, this process could be reverted with UV irradiation and, as such, it is reminiscent of **DTE1** photochromism (Figure S4).

Due to its reduced complexity, we subsequently focused on further characterizing **A1**_**open**_ from which the structure of **A1**_**closed**_ was derived. In this way, **A1**_**open**_ and **A1**_**closed**_ were proven to consist of inseparable mixtures of two and four diastereoisomeric pairs of enantiomers, respectively, whose general structures are shown in Figure 3a (see Scheme S2 for the exact stereoisomers). Specific experimental data underpin this assignment. First, a single molecular mass was determined for **A1**_**open**_ by ESI-MS, which matches the value expected for the monocycloaddition products of **DTE1** with one unreacted trifluoromethyl carbonyl group (Figure S5). This result is corroborated by the NMR spectra registered for **A1**_**open**_, which also demonstrate selective hemiacetal formation and 1,3,8-trisubstitution of the isochroman moiety generated, i.e., regio-specific cycloaddition between **DTE1**_**closed**_ and ***o***-**MBA1** (Scheme S2 and Figure S6), a typical feature of *oxo*-Diels–Alder reactions.^9^ Finally, all attempts to resolve the diastereoisomeric mixture of **A1**_**open**_ were found to be unsuccessful (e.g., by LC-MS) and, indeed, variation of the stereoisomer relative ratio was observed in time and upon treatment with acidic media (Figure S7). As previously reported for other cycloadducts formed between *o*-QDMs and trifluoromethyl ketones,^15^ this is a clear signature of mutual interconversion between these stereoisomers by hemiacetal epimerization (Figure S7).

Despite the large ***o***-**MBA1** excess used in our photoreactivity experiments with **DTE1**_**closed**_, selective UV-induced formation of the monocycloaddition adduct **A1**_**closed**_ was found, which did not further evolve into the dicycloaddition product **B1**_**closed**_. In fact, **B1**_**closed**_ could only be detected when exposing the reaction mixture to very long irradiation times (>10 h) that also led to photodegradation (Figure S8). Therefore, this result evidences that the rate of the *oxo*-Diels–Alder process critically decreases when converting one of the trifluoromethyl carbonyl groups of **DTE1**_**closed**_ into a hemiacetal moiety, i.e., by removing the electron-withdrawing effect imparted on the other reactive trifluoromethyl carbonyl group. To confirm our conclusion, we prepared monotrifluoromethyl ketone **2** (Scheme S3) and tested its light-induced reactivity with ***o***-**MBA1** under equivalent conditions as for **DTE1**_**closed**_ (Figure 4a). Clearly, no cycloadduct formation was observed in this case, which further corroborates that fast *oxo*-Diels–Alder reactivity of trifluoromethyl ketones with *o*-QDMs at ambient conditions requires activation by effective conjugation to the electron-withdrawing groups (EWGs).

**Figure 4:**
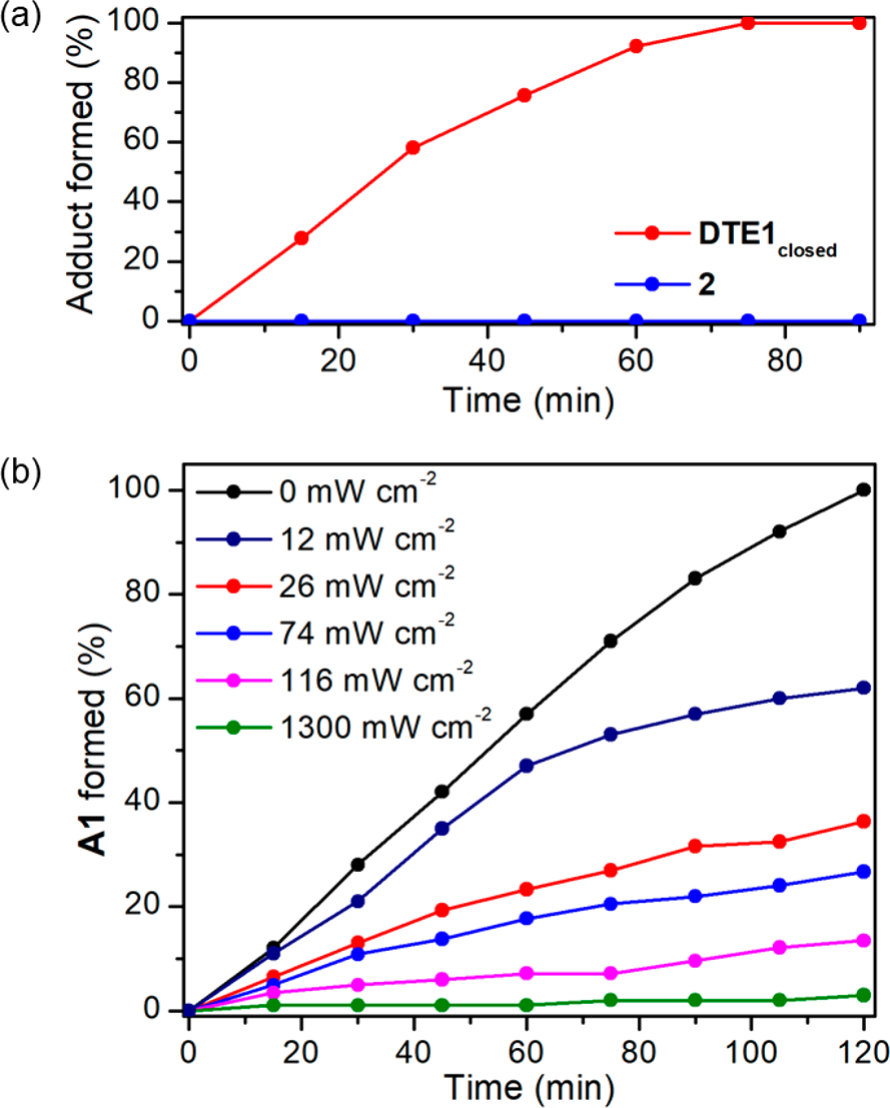
(a) Formation rate of the adduct produced by *oxo*-Diels–Alder cycloaddition of the diene photogenerated from ***o***-**MBA1** under UV illumination (LED λ_max_ = 365 nm, 0.017 mW cm^–2^) and two trifluoromethyl ketones in toluene: **DTE1**_**closed**_ and model trifluoromethylthienylketone **2** (Scheme S3). (b) Two-color modulation of the formation rate of the *oxo*-Diels–Alder cycloadduct formed by the reaction between **DTE1**_**open**_ and ***o***-**MBA1** in toluene upon simultaneous irradiation with constant UV intensity (LED λ_max_ = 365 nm, 0.017 mW cm^–2^) and variable visible light power density (LED λ_max_ = 625 nm; 0 to 1300 mW cm^–2^).

In the case of **DTE1**_**closed**_, such an activation effect could just be inhibited by photoisomerization into **DTE1**_**open**_ with visible light, where the two side trifluoromethyl carbonyl groups are no longer electronically connected (Figure 1c). In this way, we could reach visible light-induced control of the UV-triggered *oxo*-Diels–Alder reaction between **DTE1** and ***o***-**MBA1**. To demonstrate this concept, we monitored the evolution of the cycloaddition process between **DTE1**_**open**_ and ***o***-**MBA1** at ambient temperature under simultaneous illumination with UV (LED λ_max_ = 365 nm) and visible (LED λ_max_ = 625 nm) light (Figure 4b). In the absence of visible light, full conversion of **DTE1**_**open**_ into adduct products (92:8 **A1**_**closed**_/**A1**_**open**_ mixture) was observed after 120 min, though with a somewhat slower rate than when ***o***-**MBA1** was directly reacted with **DTE1**_**closed**_. Hence, efficient UV-induced ligation occurred even when starting from the open isomer of **DTE1**, as it rapidly photoisomerizes into the activated closed state. In contrast, a dramatic reduction in photoreactivity was observed when the reaction mixture was concomitantly irradiated with red light of increasing intensity (LED λ_max_ = 625 nm). Since none of the initial reagents (**DTE1**_**open**_ and ***o***-**MBA1**) absorb red light, its effect on the *oxo*-Diels–Alder reaction is exclusively attributed to the photoactivity of red-absorbing **DTE1**_**closed**_. In particular, illumination with visible light converts the UV-generated, activated dienophile **DTE1**_**closed**_ back to **DTE1**_**open**_ (Figure S9) and, accordingly, it must decrease the cycloaddition process with the photoinduced ***o***-**QMD1** diene. Indeed, the fact that this reaction was virtually suppressed under sufficiently intense red illumination at which **DTE1**_**closed**_ concentration is nearly residual proves that the *oxo*-Diels–Alder cycloaddition rate for the open isomer is negligible at ambient temperature. Therefore, our results unambiguously demonstrate light-induced modulation of **DTE1** reactivity with *o*-QMDs and, as such, the viability of our dual-wavelength gated photoligation strategy; i.e., whereas UV irradiation triggers the *oxo*-Diels–Alder reaction by concurrently generating the reactive **DTE1**_**closed**_ and ***o***-**QDM1**, illumination with visible light halts the process by photoisomerizing **DTE1**_**closed**_ back to inactive **DTE1**_**open**_.

In summary, we herein introduce a dual-color controlled photoligation process that relies on the *oxo*-Diels–Alder reaction between two light-responsive compounds. Specifically, λ_1_ (365 nm) induces the activation of a photocaged diene, while λ_2_ (625 nm) regulates the reactivity of a photoswitchable carbonyl ene. As a result, covalent bond formation can be effectively induced and halted on demand by λ_1_ and λ_2_. Such dual-color reaction control offers critical advantages to photoligation chemistry that cannot be reached by merely using a single photoactivating excitation source (e.g., turning on and off λ_1_). First, it allows true confinement of the photoreaction in time and space using patterned two-color illumination, which inhibits the effects of the λ_1_-photoactivated reagent diffusing out of the irradiated region or surviving longer than the photoexcitation time. Second, it may allow the reduction of the spatial resolution of the photoligation process to the nanometer scale by applying advanced lithographic techniques^6^ on the basis of on–off photoswitchable reactions. Therefore, our photochemical system can find direct application in the preparation of multiresponsive photoresists for the development of miniaturized functional structures.

## Supporting Information Available

Further details on compound synthesis and characterization and light-controlled *oxo*-Diels–Alder cycloaddition (PDF)
