# Supplementary material for: Dual-Wavelength Gated oxo-Diels–Alder Photoligation
Source: Org Lett. 2021 Feb 23;23(7):2405–10. doi: 10.1021/acs.orglett.1c00015 (PMC8483443; doi:10.1021/acs.orglett.1c00015)
Supplement: Supplementary file 1 — ol1c00015_si_001.pdf [file ol1c00015_si_001.pdf]

Supporting Information for:

Dual-Wavelength Gated *oxo*-Diels-Alder Photoligation

Marc Villabona,[a] Sandra Wiedbrauk,[b] Florian Feist,[b] Gonzalo Guirado,[a] Jordi Hernando,[a,*] and Christopher Barner-Kowollik[b,*]

[a] Departament de Química, Universitat Autònoma de Barcelona, Edifici C/n, Campus UAB, 08193 Cerdanyola del Vallès (Spain)

[b] Centre for Materials Science, School of Chemistry and Physics, Queensland University of Technology (QUT), 2 George Street, QLD 4000, Brisbane (Australia)

Table of Contents:

1. Materials and Methods **3**

2. Synthesis and Photochemical Characterization of DTE1 **5**

3. Synthesis of *o*-MBA1 **7**

4. *oxo*-Diels-Alder Photoligation between DTE1$_{open}$ and *o*-MBA1 **8**

5. *oxo*-Diels-Alder Photoligation between Model Trifluoromethyl Ketone 2 and *o*-MBA1 **18**

6. Light-Induced Modulation of the *oxo*-Diels-Alder Photoligation between DTE1 and *o*-MBA1 **19**

7. NMR Spectra **20**

8. References **26**

1. Materials and Methods

***Materials*:** 2-methylthiophene (Sigma-Aldrich), *N*-chlorosuccinimide (Sigma-Aldrich), glutaryl chloride (Sigma-Aldrich), aluminium chloride (Fisher), titanium chloride 1 M toluene (Fluorochem), zinc (Sigma-Aldrich), *tert*-butyl lithium 1.6 M in pentane (Sigma-Aldrich), ethyl trifluoroacetate (Fluorochem), 2-fluoro-6-methylbenzaldehyde (Fluorochem), butanethiol (Sigma-Aldrich), lithium diisopropylamide 2 M in THF/heptane/benzene (Sigma-Aldrich), sodium hydroxide (Fisher), potassium carbonate (Fisher), sodium hydrogencarbonate (Fisher), sodium chloride (Fisher), acetic acid (Fisher), acetonitrile (Fisher), toluene (Fisher), *N,N*-dimethylformamide (Fisher), hexane (Scharlab), ethyl acetate (Scharlab), dichloromethane (Fisher), tetrahydrofuran (Fisher), chloroform-*d* (Euroisotop), acetonitrile-d_3 (Euroisotop) and toluene-d_8 (Euroisotop) were purchased and used as received unless specified. Preparative thin layer chromatography (TLC) purifications were performed using silica gel 60 precoated aluminum plates (0.20 mm thickness). Flash column chromatography was performed using silica gel (230-400 mesh).

***Methods*:** NMR spectra were recorded on Bruker DPX250 (250 MHz for ^{1}H), DPX360 (360 MHz for ^{1}H), AvanceIII 400NB (400 MHz for ^{1}H) and Bruker System 600 Ascend LH (600 MHz for ^{1}H). The δ-scale was normalized relative to the residual solvent signal for ^{1}H NMR and ^{13}C NMR ($CDCl_3$ (7.26 ppm for ^{1}H; 77.2 ppm for ^{13}C), CD_3CN (1.94 ppm for ^{1}H; 118.3 and 1.3 ppm for ^{13}C) and C_7D_8 (7.09, 7.01, 6.97 and 2.08 ppm for ^{1}H; 137.5, 128.9, 128.0, 125.1 and 20.4 ppm for ^{13}C)) and relative to $CFCl_3$ for ^{19}F NMR (0.00 ppm in all the solvents). NMR signals for selected molecules were assigned with the help of COSY, HSQC, HMBC and DEPT135. LC-MS measurements were performed on an UltiMate 3000 UHPLC System (Dionex) consisting of a pump (LPG 3400SZ, autosampler WPS 3000TSL) and a temperature controlled column department (TCC 3000). Separation was performed on a C18 HPLC-column (Phenomenex Luna 5µm, 100 Å, 250 × 2.0 mm) operating at 40 °C. A gradient of CD_3CN:H_2O

80:20 – 100:0 v/v at a flow rate of 0.40 mL·min^{-1} during 10 min was used as the eluting solvent. The flow was split in a 9:1 ratio, where 90% of the eluent were directed through the UV-detector (VWD 3400, Dionex, detector wavelengths 215, 254, 280, 360 nm) and 10% were infused into the electrospray source. Spectra were recorded on a LTQ Orbitrap Elite mass spectrometer (ThermoFisher Scientific) equipped with an HESI II probe. The instrument was calibrated in the m/z range 74-1822 using premixed calibration solutions (Thermo Scientific). A constant spray voltage of 3.5 kV, a dimensionless sheath gas and a dimensionless auxiliary gas flow rate of 5 and 2 were applied, respectively. The capillary temperature and was set to 300 °C, the S-lens RF level was set to 68, and the aux gas heater temperature was set to 125 °C. IR-ATR spectra were recorded in a Bruker Tensor 27 Golden Gate spectrometer with a diamond tip. UV/vis spectra were recorded on a Shimadzu UV-2700 spectrophotometer equipped with a CPS-100 electronic temperature-controlled cell positioner or on a Agilent HP 8453 spectrophotometer. Samples were measured in Hellma Analytics quartz high precision cells with a path length of 10 mm at ambient temperature.

2. Synthesis and Photochemical Characterization of DTE1

Synthesis of DTE1$_{open}$: The synthesis of **DTE1$_{open}$** started from dichlorodithienylethene derivative **1**, which was prepared as previously reported by us.[1] Under inert atmosphere, 416 mg of **1** (1.26 mmol) were dissolved in 20 mL of anhydrous THF. The solution was cooled down to -78ºC and 2 mL of 1.6 M *tert*-butyl lithium solution in anhydrous pentane (3.2 mmol) were added. After 15 min of stirring, the organolithium compound was quenched with 2 mL of ethyl trifluoroacetate (13 mmol) and the reaction was stirred at room temperature for 30 min. 20 mL of water were added and the mixture was extracted twice with 20 mL of diethyl ether. The organic layers were combined, dried using anhydrous $MgSO_4$ and the solvent removed under vacuum. **DTE1$_{open}$** was obtained as a brownish oil (340.1 mg, 0.76 mmol, 60% yield) after flash column chromatography (Cyclohexane:EtOAc, 19:1). ^{1}H NMR (400 MHz, $CDCl_3$) δ= 7.59 (s, 2H), 2.87 (t, *J* = 7.5 Hz, 4H), 2.22 – 2.12 ppm (m, 8H); ^{13}C NMR (101 MHz, $CDCl_3$) δ= 173.0 (q, *J* = 36.6 Hz), 149.9, 138.0, 137.6 (q, *J* = 3.2 Hz), 135.6, 132.6, 116.5 (q, *J* = 290.6 Hz), 38.3, 23.0, 15.4 ppm; ^{19}F NMR (376 MHz, $CDCl_3$) δ= -72.68 ppm (s); IR (ATR, cm^{-1}): 2956, 2848, 2325, 2051, 1681, 1529, 1424, 1373, 1333, 1234, 1194, 1136, 1028, 925, 868, 754, 738, 719, 676, 636; HRMS (ESI) m/z: [M-H]$^-$ Calcd for $C_{19}H_{13}F_6O_2S_2^-$ 451.0267; Found 451.0261. See Figures S10, S11 and S12 for the ^{1}H, ^{13}C and ^{19}F NMR spectra of **DTE1$_{open}$**, respectively.

Synthesis of DTE1$_{closed}$: 30 mg of **DTE1$_{open}$** in 5 mL of acetonitrile were irradiated for 5 h with a LED λ_{max} = 365 nm (23 mW cm^{-2}). The solvent was then removed in vacuo and the product purified by preparative TLC (hexanes:EtOAc 9:1) to obtain 17.5 mg of **DTE1$_{closed}$** as a dark blue powder (58% yield). ^{1}H NMR (250 MHz, CD_3CN): δ= 7.11 (s, 2H), 2.61 (t, *J* = 7.4 Hz, 4H), 2.02 ppm (s, 6H), 1.96-1.90 ppm (m, 2H); ^{13}C NMR (101 MHz, CD_3CN) δ= 176.2 (q, *J* = 36.7 Hz), 149.5, 146.7, 142.3, 137.9 (q, *J* = 260.5 Hz), 134.5 (q, *J* = 3.7 Hz), 65.3, 31.3, 28.3, 25.4 ppm; ^{19}F NMR (235 MHz, CD_3CN): δ= -71.26 ppm (s); IR (ATR, cm^{-1}): 2929, 1667, 1617,

1499, 1437, 1375, 1339, 1300, 1188, 1127, 897, 842, 752, 733, 682, 641; HRMS (ESI) m/z: $[M-H]^-$ Calcd for $C_{19}H_{13}F_6O_2S_2^-$ 451.0267; Found 451.0271. See Figures S13, S14 and S15 for the ^1H, ^{13}C and ^{19}F NMR spectra of **DTE1$_{closed}$**, respectively.

Determination of photoisomerization quantum yields: Photoisomerization quantum yields of the two isomers of **DTE1** were determined using the methodology reported in ref. 2 and 1,2-bis(5-chloro-2-methyl-3-thienyl)perfluorocyclopentene in hexane as reference compound (Φ_{o-c}= 0.47 and Φ_{c-o}= 0.13).[3] For the ring-closing process, the third harmonic of a ns-pulsed Nd:YAG laser (λ_{exc} = 355 nm) was used as excitation source, while a cw laser diode at λ_{exc} = 532 nm was employed to evaluate the quantum yield of the ring-opening reaction.

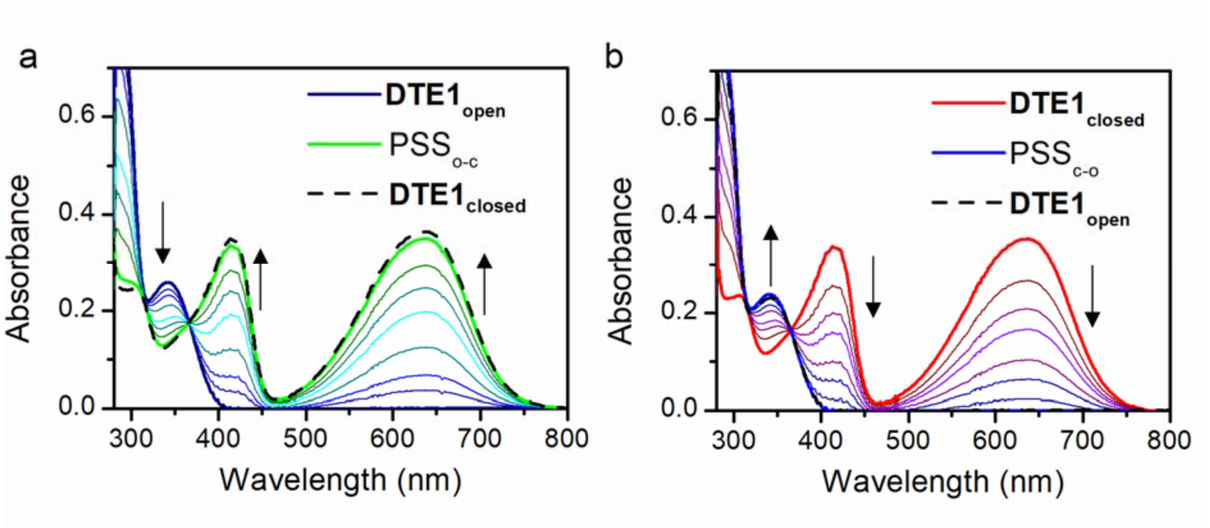

Figure S1. (a) Variation of the absorption spectrum of **DTE1$_{open}$** in toluene (c = 3.5·10^{-5} M) when irradiated at λ_{exc} = 365 nm until a photostationary state is obtained (PSS$_{o-c}$). For sake of comparison, the spectrum of **DTE1$_{closed}$** is also shown. (b) Variation of the absorption spectrum of **DTE1$_{closed}$** in toluene (c = 3.5·10^{-5} M) when irradiated at λ_{exc} = 650 nm until a photostationary state is obtained (PSS$_{c-o}$). For sake of comparison, the spectrum of **DTE1$_{open}$** is also shown.

3. Synthesis of *o*-MBA1

***Synthesis of o-MBA1*:** A dry round-bottom flask was charged with K_2CO_3 (1.041 mg, 7.53 mmol), 2-fluoro-6-methylbenzaldehyde (0.5 mL, 4.17 mmol), and 1-butanethiol (0.60 mL, 5.60 mmol) and the mixture was dissolved in 20 mL of dry DMF under an argon atmosphere. The reaction mixture was heated to 85 °C for 12 h. Next, the reaction mixture was cooled to room temperature and dissolved in 75 mL of ethyl acetate, and the organic phase was washed with 50 mL of water, 2x25 mL of brine and dried over Na_2SO_4. After a flash column cromathography in hexanes, the product was obtained as 0.856 g of a yellowish oil (4.14 mmol, 98% yield). Spectral data of ***o*-MBA1** was in agreeement with that previously reported by us.[4] ^{1}H NMR (360 MHz, $CDCl_3$) δ= 10.69 (s, 1H), 7.35 (t, J = 7.7 Hz, 1H), 7.29 (d, J = 7.9 Hz, 1H), 7.04 (d, J = 7.3 Hz, 1H), 2.91 (t, J = 7.4 Hz, 2H), 2.60 (s, 3H), 1.66 (p, J = 7.3 Hz, 2H), 1.47 (h, J = 7.3 Hz, 2H), 0.93 (t, J = 7.3 Hz, 3H) ppm. ^{13}C NMR (151 MHz, $CDCl_3$) δ= 192.7, 143.0, 142.1, 132.9, 132.7, 129.0, 126.9, 33.9, 30.8, 22.2, 20.8, 13.8 ppm.

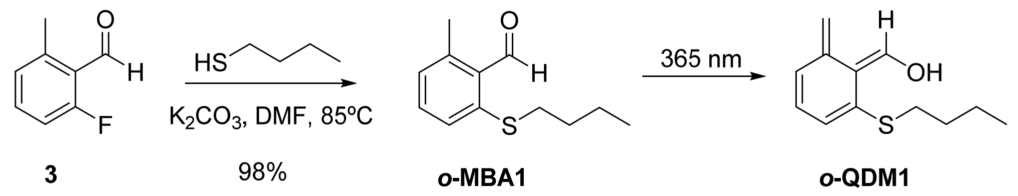

Scheme S1. Synthesis of ***o*-MBA1**. Under irradiation with UV light, ***o*-MBA1** converts into the activated diene ***o*-QDM1**.

4. *oxo*-Diels-Alder Photoligation between $\textbf{DTE1}_{\textbf{open}}$ and *o*-MBA1

***Procedure of the Photoligation Reaction*:** A 20 mL vial containing 10.2 mg of ***o*-MBA1** (0.049 mmol) and 19.9 mg of $\textbf{DTE}_{\textbf{open}}$ (0.043 mmol) in 15 mL of degassed acetonitrile was irradiated overnight using a LED λ_{max} = 365 nm (23 mW cm^{-2}). After reaction completion, the crude was irradiated for 15 min with a LED λ_{max} = 625 nm (100 mW cm^{-2}). The solvent was removed and the product purified by preparative TLC (hexanes:EtOAc 9:1) obtaining 20.4 mg of $\textbf{A1}_{\textbf{open}}$ (0.030 mmol, 70%) as a brownish solid corresponding to a mixture of the two diastereomeric pairs of enantiomers $\textbf{A1a}_{\textbf{open}}$ and $\textbf{A1b}_{\textbf{open}}$ in a 87:13 ratio. ^{1}H NMR (600 MHz, CD_3CN) δ= 7.72 – 7.68 (m, 1H, $\textbf{A1a}_{\textbf{open}}$, 1H, $\textbf{A1b}_{\textbf{open}}$), 7.36 (d, *J* = 8.1 Hz, 1H, $\textbf{A1b}_{\textbf{open}}$), 7.33 (d, *J* = 8.0 Hz, 1H, $\textbf{A1a}_{\textbf{open}}$), 7.30 (t, *J* = 7.8 Hz, 1H, $\textbf{A1b}_{\textbf{open}}$), 7.22 (t, *J* = 7.7 Hz, 1H, $\textbf{A1a}_{\textbf{open}}$), 7.13 (d, *J* = 7.4 Hz, 1H, $\textbf{A1b}_{\textbf{open}}$), 7.00 (d, *J* = 7.5 Hz, 1H, $\textbf{A1a}_{\textbf{open}}$), 6.86 (s, 1H, $\textbf{A1b}_{\textbf{open}}$), 6.71 (s, 1H, $\textbf{A1a}_{\textbf{open}}$), 6.59 (d, *J* = 4.8 Hz, 1H, $\textbf{A1b}_{\textbf{open}}$), 6.58 (d, *J* = 4.8 Hz, 1H, $\textbf{A1a}_{\textbf{open}}$), 5.02 (d, *J* = 4.9 Hz, 1H, $\textbf{A1a}_{\textbf{open}}$), 4.98 (d, *J* = 4.9 Hz, 1H, $\textbf{A1b}_{\textbf{open}}$), 3.71 (d, *J* = 14.6 Hz, 1H, $\textbf{A1a}_{\textbf{open}}$), 3.54 (s, 2H, $\textbf{A1b}_{\textbf{open}}$), 3.26 (d, *J* = 14.5 Hz, 1H, $\textbf{A1a}_{\textbf{open}}$), 2.97 – 2.79 (m, 2H, $\textbf{A1a}_{\textbf{open}}$, 2H, $\textbf{A1b}_{\textbf{open}}$), 2.78 – 2.65 (m, 2H, $\textbf{A1a}_{\textbf{open}}$, 2H, $\textbf{A1b}_{\textbf{open}}$), 2.11 – 2.06 (m, 5H, $\textbf{A1b}_{\textbf{open}}$), 2.02 (p, *J* = 7.6 Hz, 2H, $\textbf{A1a}_{\textbf{open}}$), 2.01 (s, 3H, $\textbf{A1b}_{\textbf{open}}$), 1.80 (s, 3H, $\textbf{A1a}_{\textbf{open}}$), 1.68 (s, 3H, $\textbf{A1a}_{\textbf{open}}$), 1.57 – 1.51 (m, 2H, $\textbf{A1b}_{\textbf{open}}$), 1.49 (q, *J* = 7.1 Hz, 1H, $\textbf{A1a}_{\textbf{open}}$), 1.43 – 1.33 (m, 2H, $\textbf{A1a}_{\textbf{open}}$, 2H, $\textbf{A1b}_{\textbf{open}}$), 0.89 (t, *J* = 7.4 Hz, 3H, $\textbf{A1b}_{\textbf{open}}$), 0.86 ppm (t, *J* = 7.3 Hz, 3H, $\textbf{A1a}_{\textbf{open}}$); ^{13}C NMR (151 MHz, CD_3CN) δ= 175.8 (q, *J* = 54.9 Hz, $\textbf{A1b}_{\textbf{open}}$), 173.5 (q, *J* = 36.1 Hz, $\textbf{A1a}_{\textbf{open}}$), 151.6 ($\textbf{A1a}_{\textbf{open}}$), 141.4 ($\textbf{A1b}_{\textbf{open}}$), 140.1 ($\textbf{A1b}_{\textbf{open}}$), 140.0 ($\textbf{A1a}_{\textbf{open}}$), 139.5 (q, *J* = 2.7 Hz, $\textbf{A1b}_{\textbf{open}}$), 139.2 (q, *J* = 2.7 Hz, $\textbf{A1a}_{\textbf{open}}$), 138.9 ($\textbf{A1a}_{\textbf{open}}$), 138.3 ($\textbf{A1b}_{\textbf{open}}$), 137.9 ($\textbf{A1a}_{\textbf{open}}$), 137.0 ($\textbf{A1b}_{\textbf{open}}$), 136.8 ($\textbf{A1a}_{\textbf{open}}$), 136.6 ($\textbf{A1a}_{\textbf{open}}$), 136.4 ($\textbf{A1b}_{\textbf{open}}$), 136.3 ($\textbf{A1a}_{\textbf{open}}$), 135.9 ($\textbf{A1b}_{\textbf{open}}$), 134.3 ($\textbf{A1a}_{\textbf{open}}$), 134.3 ($\textbf{A1b}_{\textbf{open}}$), 134.0 ($\textbf{A1b}_{\textbf{open}}$), 134.0 ($\textbf{A1a}_{\textbf{open}}$), 133.8 ($\textbf{A1b}_{\textbf{open}}$), 132.7 ($\textbf{A1a}_{\textbf{open}}$), 132.7 ($\textbf{A1b}_{\textbf{open}}$), 132.4 ($\textbf{A1a}_{\textbf{open}}$), 130.9 ($\textbf{A1b}_{\textbf{open}}$), 130.7 ($\textbf{A1a}_{\textbf{open}}$), 130.3 ($\textbf{A1b}_{\textbf{open}}$), 130.2 ($\textbf{A1a}_{\textbf{open}}$), 129.9 ($\textbf{A1b}_{\textbf{open}}$), 128.0 ($\textbf{A1a}_{\textbf{open}}$), 127.6 ($\textbf{A1a}_{\textbf{open}}$), 127.3 ($\textbf{A1b}_{\textbf{open}}$), 126.7 ($\textbf{A1b}_{\textbf{open}}$), 125.7 (q, *J* = 276.5 Hz, $\textbf{A1b}_{\textbf{open}}$), 125.4 (q, *J* = 283.3 Hz, $\textbf{A1a}_{\textbf{open}}$), 117.5 (q, *J* = 289.8 Hz, $\textbf{A1a}_{\textbf{open}}$), 92.2

(**A1b**$_{open}$), 91.7 (**A1a**$_{open}$), 79.4 (q, J = 30.2 Hz, **A1a**$_{open}$), 77.6 (q, J = 29.1 Hz, **A1b**$_{open}$), 38.9 (**A1a**$_{open}$), 38.8 (**A1a**$_{open}$), 38.8 (**A1b**$_{open}$), 38.5 (**A1b**$_{open}$), 35.0 (**A1a**$_{open}$), 34.7 (**A1b**$_{open}$), 34.3 (**A1b**$_{open}$), 33.3 (**A1a**$_{open}$), 32.0 (**A1a**$_{open}$), 32.0 (**A1b**$_{open}$), 27.6 (**A1b**$_{open}$), 23.8 (**A1b**$_{open}$), 23.6 (**A1a**$_{open}$), 22.5 (**A1a**$_{open}$), 15.4 (**A1b**$_{open}$), 15.3 (**A1a**$_{open}$), 14.2 (**A1b**$_{open}$), 14.1 (**A1a**$_{open}$), 14.0 (**A1a**$_{open}$), 13.9 ppm (**A1b**$_{open}$); ^{19}F NMR (565 MHz, CD_3CN) δ= -71.57 (d, J = 1.6 Hz, 3F, **A1b**$_{open}$), -71.60 (d, J = 1.6 Hz, 3F, **A1a**$_{open}$), -78.90 (s, 3F, **A1b**$_{open}$), -79.80 ppm (s, 3F, **A1a**$_{open}$); IR (ATR, cm^{-1}): 2929, 1735, 1681, 1649, 1583, 1560, 1534, 1437, 1334, 1289, 1267, 1227, 1142, 1024, 984, 869, 843, 755, 718, 681, 662, 628; HRMS (ESI) m/z: [M-H]$^-$ Calcd for $C_{31}H_{29}F_6O_3S_3^-$ 659.1183; Found 659.1188. See Figures S16, S17 and S18 for the ^{1}H, ^{13}C and ^{19}F NMR spectra of **A1**$_{open}$, respectively.

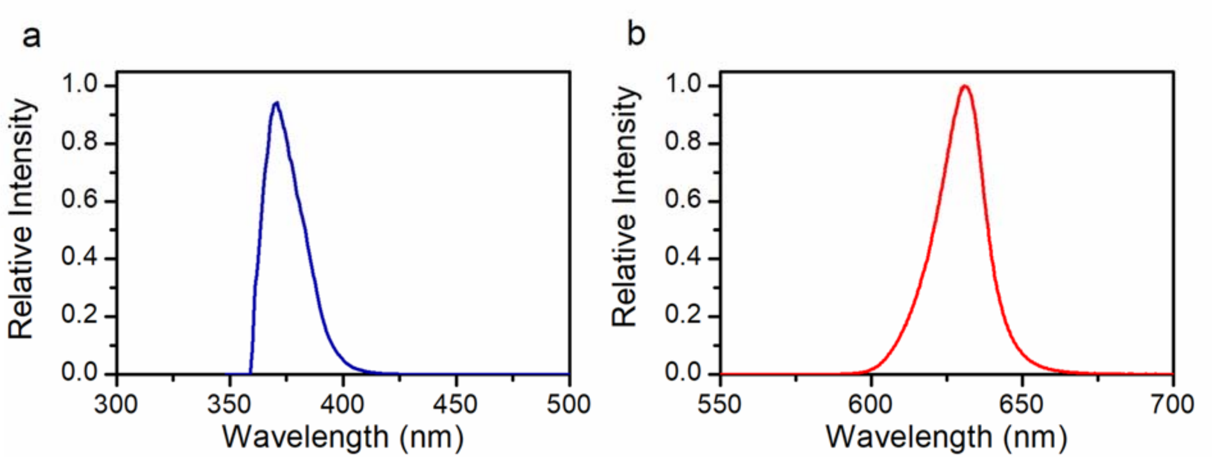

Figure S2. Emission spectra of the different LED sources used in this work to control *oxo*-Diels-Alder photoligation reactions (3 W LED λ_{max} = 365 nm, 100 W LED λ_{max} = 625 nm).

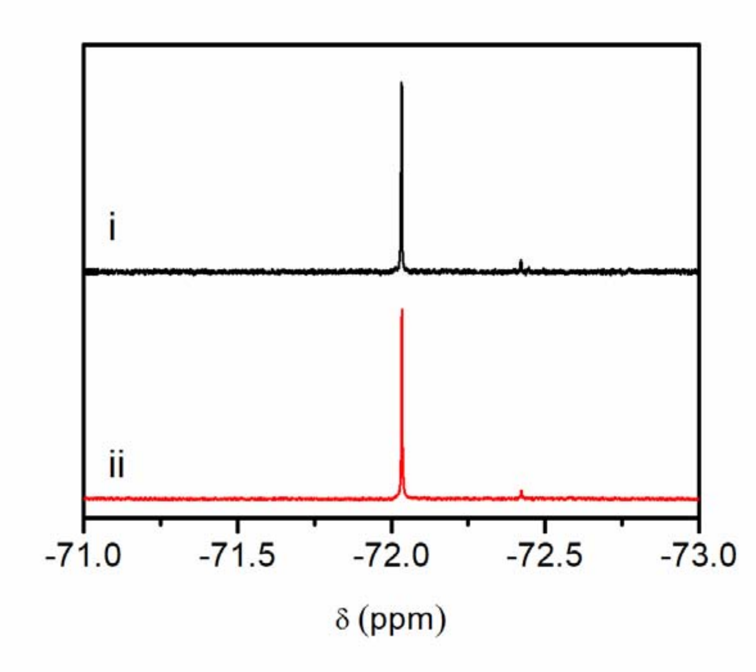

Figure S3. ^{19}F NMR spectra of (565 MHz, toluene-d_8): (i) **DTE1**$_{\mathbf{closed}}$; (ii) **DTE1**$_{\mathbf{closed}}$ after UV irradiation (LED λ_{max} = 365 nm, 0.017 mW cm^{-2}) for 360 min. The low intensity peak at δ = -72.42 ppm corresponds to the residual amount of the open isomer left when initially preparing **DTE1**$_{\mathbf{closed}}$ by photoisomerization of **DTE1**$_{\mathbf{open}}$ (96:4 **DTE1**$_{\mathbf{closed}}$:**DTE1**$_{\mathbf{open}}$ ratio for the photostationary state generated in toluene-d_8 by irradiation with a LED λ_{max} = 365 nm).

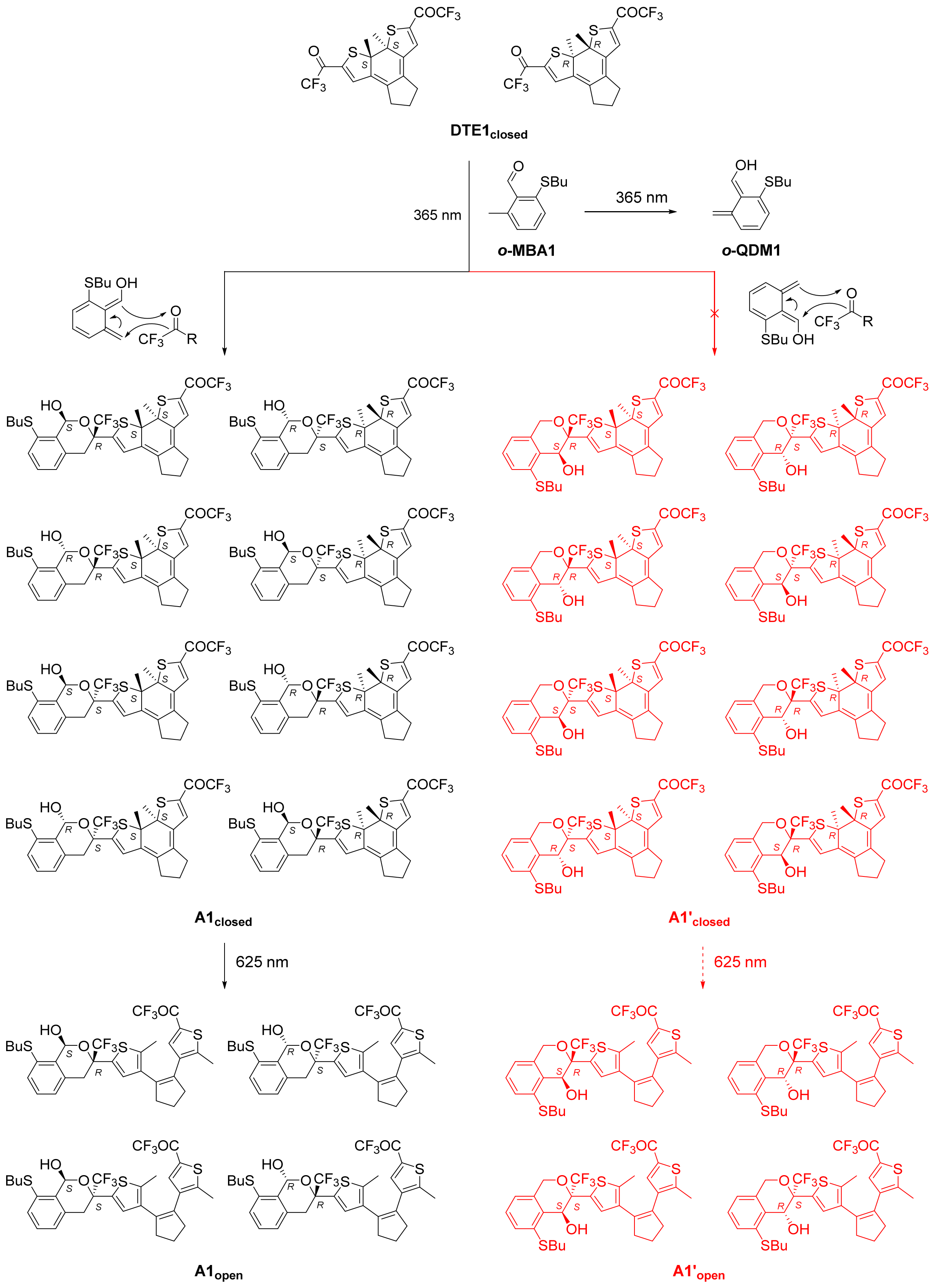

Scheme S2. Structures of all the possible adducts that could be formed by UV-induced *oxo*-Diels-Alder reaction between **DTE1**$_{\text{closed}}$ and ***o*-MBA1** (**A1**$_{\text{closed}}$ and **A1'**$_{\text{closed}}$) as well as of the corresponding open isomers obtained by photoisomerization of these products with visible light

(**A1$_{open}$** and **A1'$_{open}$**). For this, we took into account that the starting **DTE1$_{closed}$** is already composed of a mixture of two enantiomers with relative *anti* configuration of their methyl groups because the photoinduced 6π electrocyclization reaction of **DTE1$_{open}$** takes place in a conrotatory fashion.[5] In agreement with previous reports of *oxo*-Diels-Alder cycloadditions,[6–8] the regioisomers **A1'$_{closed}$**, and therefore, **A1'$_{open}$** were not obtained. Consequently, only **A1$_{closed}$** and **A1$_{open}$** were produced as inseparable mixtures of four and two diastereoisomeric pairs of enantiomers, respectively. This explains why four and two separate sets of signals were observed in the ^{19}F NMR spectra of **A1$_{closed}$** and **A1$_{open}$**, respectively (see Figure 3b and the main text). In the case of **A1$_{closed}$**, the ^{19}F NMR signals of the trifluoromethyl carbonyl group of two pairs of enantiomers overlap at δ = -70.87 ppm and they could not be resolved.

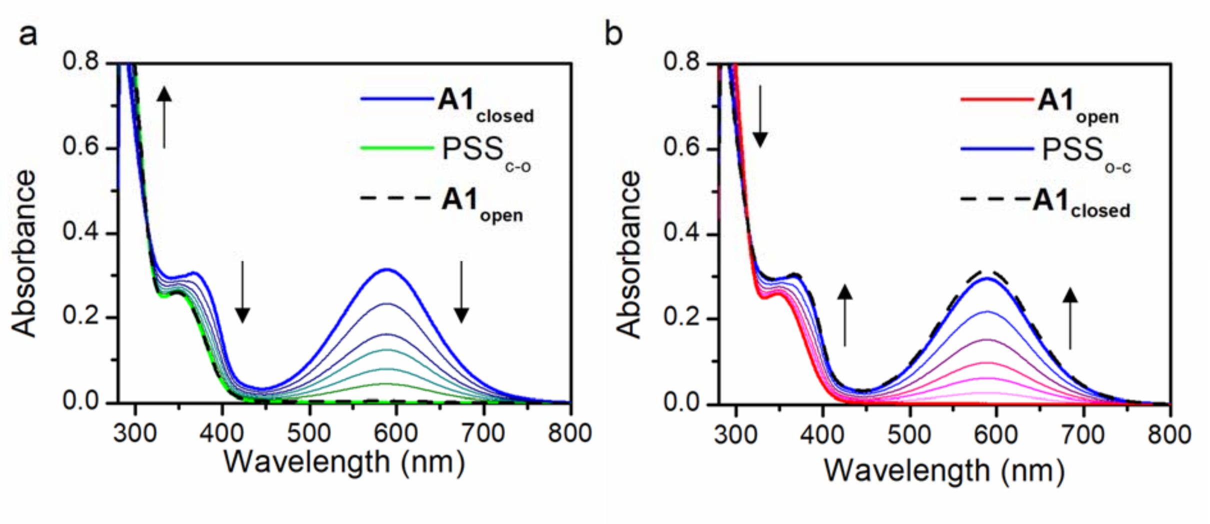

Figure S4. (a) Variation of the absorption spectrum of **A1$_{closed}$** in toluene (c = 4.0·10^{-5} M) when irradiated at λ_{exc} = 650 nm until a photostationary state is obtained (PSS$_{c-o}$). For sake of comparison, the spectrum of **A1$_{open}$** is also shown. (b) Variation of the absorption spectrum of **A1$_{open}$** in toluene (c = 4.0·10^{-5} M) when irradiated at λ_{exc} = 365 nm until a photostationary state is obtained (PSS$_{o-c}$). For sake of comparison, the spectrum of **A1$_{closed}$** is also shown.

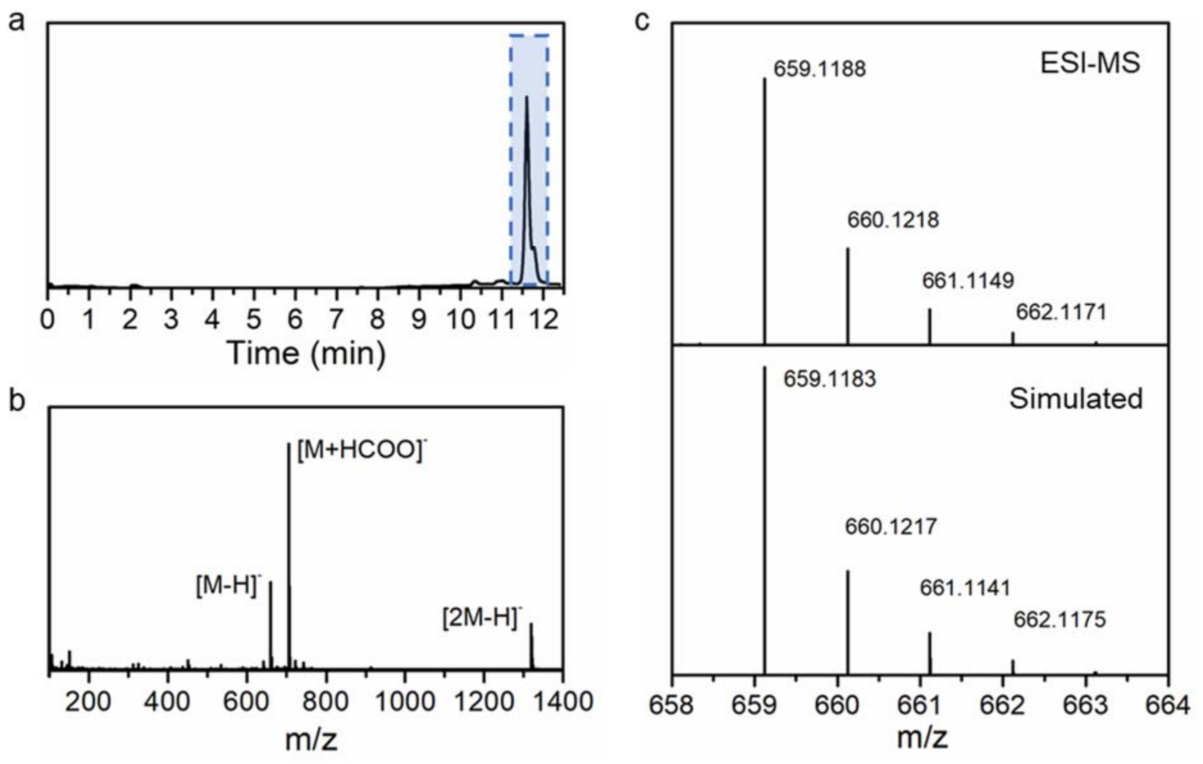

Figure S5. (a) LC trace (254 nm detector wavelength) measured for the **A1**$_{\mathbf{open}}$ product isolated from the reaction mixture obtained by UV irradiation of a 30:1 mixture of ***o*-MBA1** and **DTE1**$_{\mathbf{closed}}$ in CD_3CN for 90 min (LED λ_{max} = 365 nm**,** 0.017 mW cm^{-2}) followed by 2 min illumination with red light (LED λ_{max} = 625 nm, 35 mW cm^{-2}). (b) Mass spectrum (ESI-MS) of the main peak eluted at t = 11.6 min in (a), which together with other spectral data allowed assignment to cycloadduct **A1**$_{\mathbf{open}}$ (m/z: [M-H]$^-$ Calcd for $C_{31}H_{29}F_6O_3S_3^-$ 659.1183; Found 659.1188). (b) Comparison between the experimental (top) and simulated (bottom) isotopic pattern of the quasi-molecular peak [M-H]$^-$ detected for **A1**$_{\mathbf{open}}$ in the ESI-MS spectrum shown in (b).

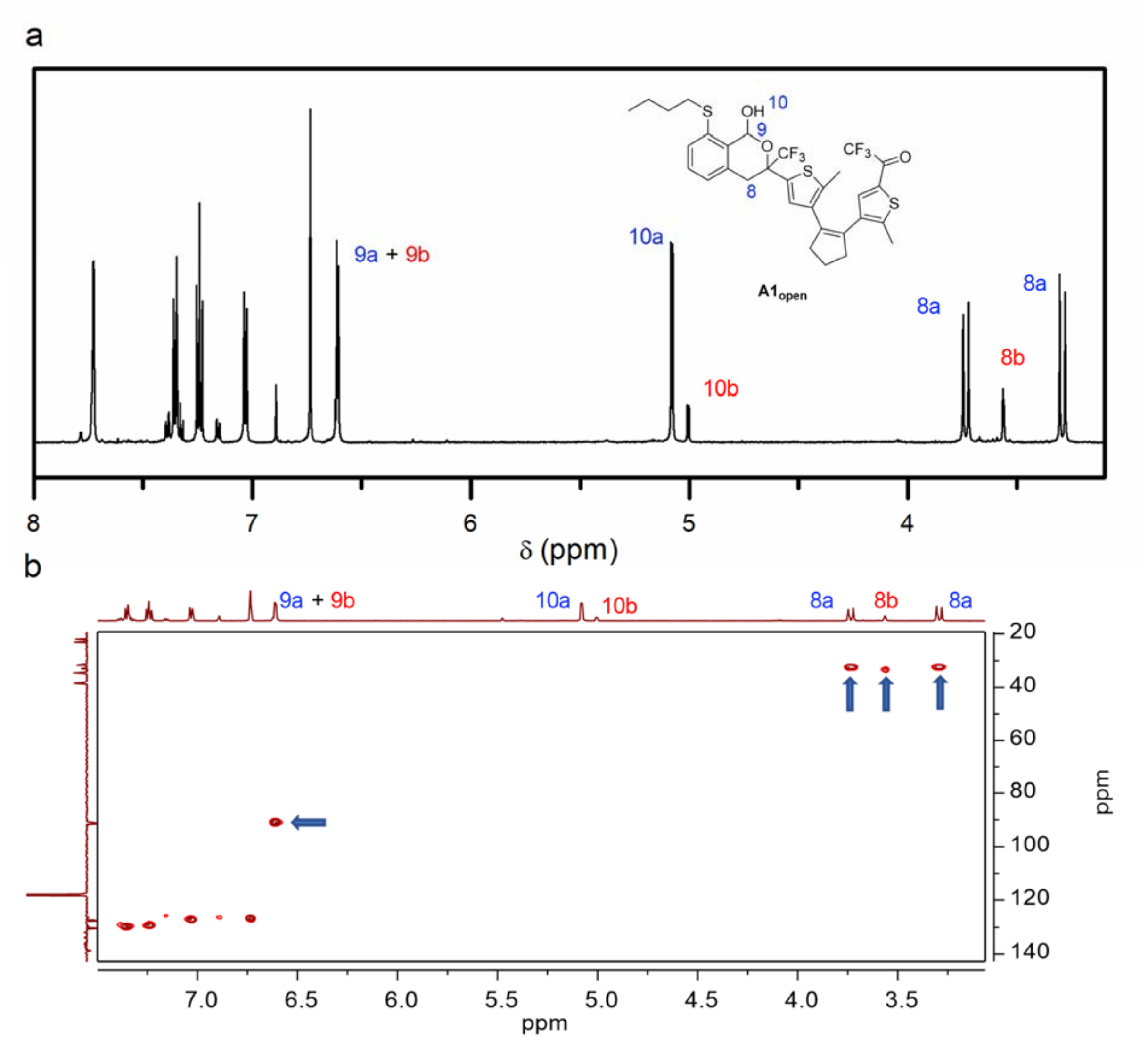

Figure S6. (a) ^{1}H NMR and (b) ^{1}H-^{13}C HSQC NMR of **A1$_{open}$** (600 MHz, CD_3CN). Considering chemical shifts and HSQC cross peaks, H8 and H9 were assigned respectively to the carbon nuclei appearing at 33.3 and 91.7 ppm for **A1a$_{open}$** and 34.3 and 92.2 ppm for **A1b$_{open}$**. The upfield carbon shift for both C8, together with the downfield carbon shift for both C9 suggest that: (a) each set of signals do not belong to different regioisomers, but to different stereoisomers of the same regioisomer; (b) C8 is not bonded to any heteroatom; and (c) C9 is forming a hemiacetal group. This leads to the general structure proposed for the adduct resulting from the *oxo*-Diels Alder reaction between **DTE1** and ***o*-MBA1** as the only possible for both **A1a$_{open}$** and **A1b$_{open}$**.

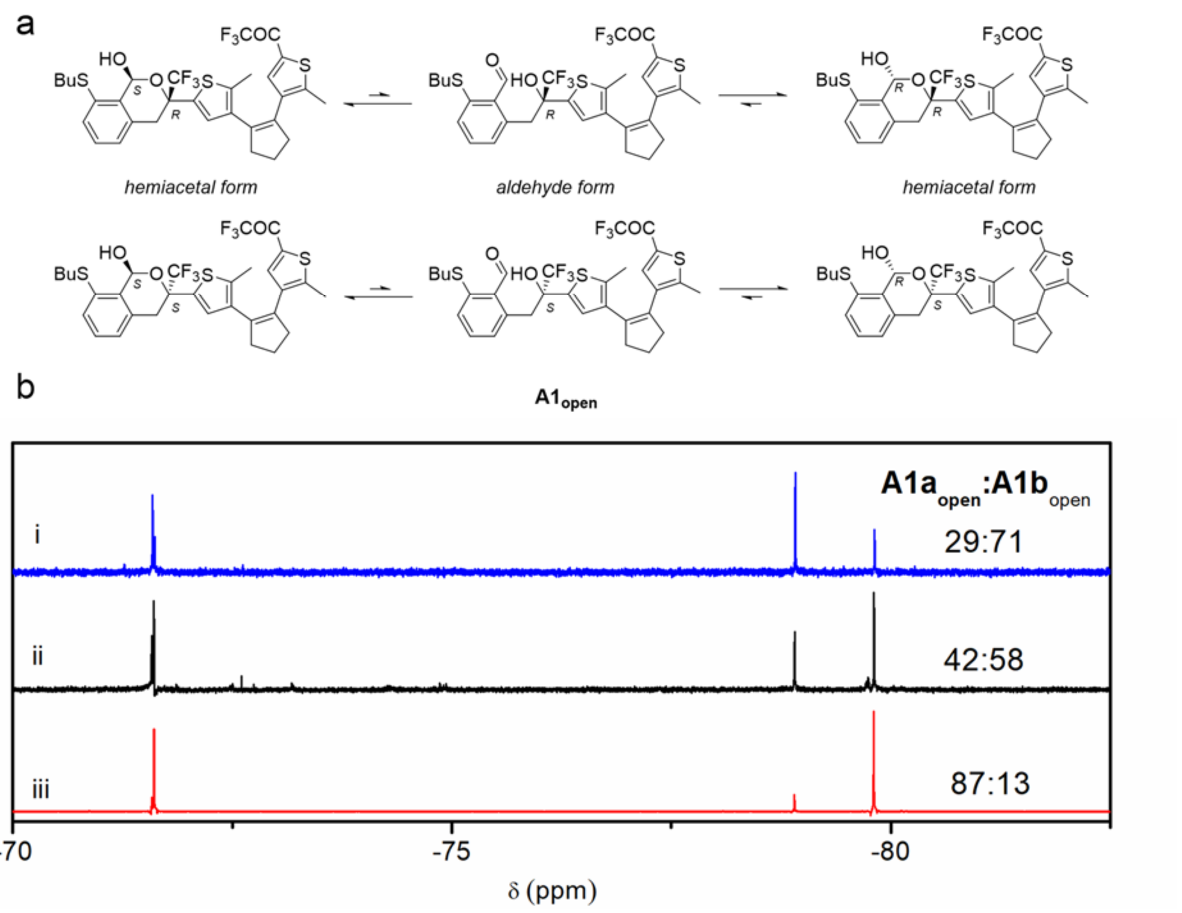

Figure S7. (a) Epimerization of $\mathbf{A1_{open}}$ by hemiacetal-aldehyde tautomerism, as previously reported for the cycloadducts formed from other trifluoromethyl ketones and *o*-QDMs.[9] No NMR signals of the aldehyde species were observed, which indicates that the tautomerization equilibria are completely displaced towards the hemiacetal forms in our case. (b) ^{19}F NMR spectra (565 MHz, CD_3CN) of $\mathbf{A1_{open}}$: (i) right after photochemical preparation; (ii) 30 min later after photochemical preparation; (iii) after filtration through silica.

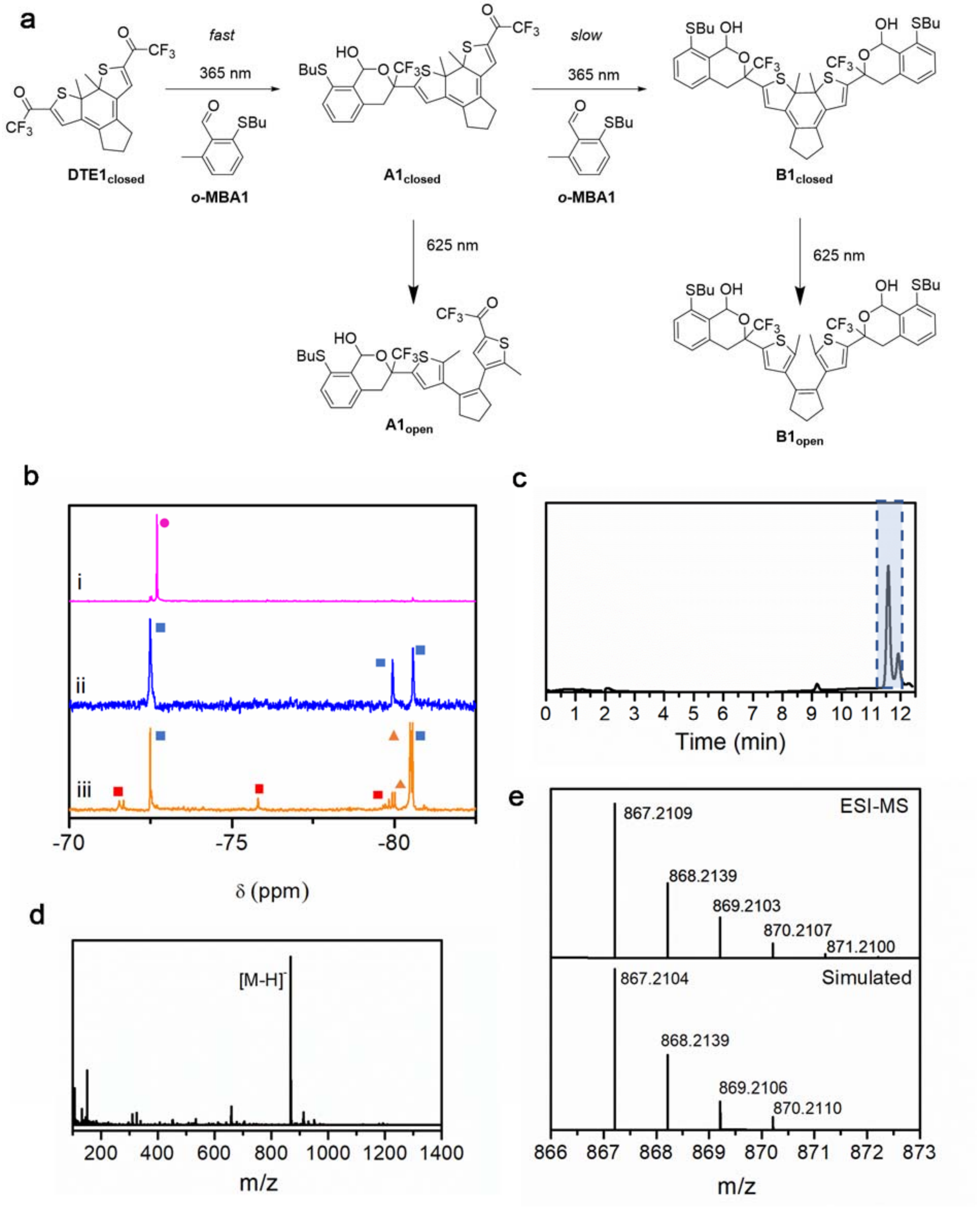

Figure S8. (a) Formation of adduct **B1$_{\mathbf{closed}}$** by double *oxo*-Diels-Alder cycloaddition on both trifluoromethyl carbonyl groups of **DTE1$_{\mathbf{closed}}$** when mixed at large concentrations with an excess of ***o*-MBA1** and irradiated with UV light for a prolonged period. (b) ^{19}F NMR spectra (565 MHz, toluene-d_8) of: (i) **DTE1$_{\mathbf{open}}$**; (ii) a mixture of **DTE1$_{\mathbf{open}}$** (c = 0.16 mM) and ***o*-MBA1** (c = 5 mM) under UV illumination (LED λ_{max} = 365 nm, 0.017 mW cm^{-2}) for 2 hours in toluene

followed by 10 min under visible light (LED λ_{max} = 625 nm, 10 mW cm^{-2}), which resulted in quantitative transformation into **A1$_{open}$**; and (iii) a mixture of **DTE1$_{open}$** (c = 0.16 mM) and ***o*-MBA1** (c = 5 mM) under UV illumination (LED λ_{max} = 365 nm, 0.017 mW cm^{-2}) for 12 hours in toluene followed by 10 min of visible light (LED λ_{max} = 625 nm, 10 mW cm^{-2}), which resulted in the formation of an appreciable amount of **B1$_{open}$** as a side product. For sake of clarity, the ^{19}F NMR signals in each spectrum arising from **DTE1$_{open}$** (pink circles), **A1$_{open}$** (blue squares) and **B1$_{open}$** (orange triangles) are indicated with different symbols. Other peaks not assigned in (iii) were tentatively attributed to photodegradation products (red squares). (c) LC trace (254 nm detector wavelength) measured for the **B1$_{open}$** product isolated from the final reaction mixture (iii) shown in (b) by preparative TLC (hexanes:EtOAc 9:1). The two peaks eluted at t = 11.6 and 11.9 min showed the same molecular mass in ESI-MS and were assigned to two different sets of stereoisomers of the dicycloaddition product **B1$_{open}$**. (d) Mass spectrum (ESI-MS) of peak eluted at t = 11.6 min in the LC chromatogram shown in (c), which was assigned to adduct **B1$_{open}$** (m/z: [M-H]$^-$ Calcd for $C_{43}H_{45}F_6O_4S_4^-$ 867.2104; Found 867.2109). (e) Comparison between the experimental (top) and simulated (bottom) isotopic pattern of the quasi-molecular peak [M-H]$^-$ registered for **B1$_{open}$** in (d).

5. *oxo*-Diels-Alder photoligation between model trifluoromethyl ketone 2 and *o*-MBA1

***Synthesis of 2*:** Compound **2** was obtained using a different method than reported in the literature.[10] Under inert atmosphere 0.5 mL of 2-methylthiophene (5 mmol) were dissolved in 20 mL of anhydrous THF. The solution was cooled down to -78°C and 3 mL of 2 M LDA (6 mmol) were added. The reaction was stirred for 15 min before adding 0.8 mL of ethyl trifluoroacetate (6.7 mmol). After stirring at room temperature for 30 min the reaction was quenched with 15 mL of brine and the organic phase was separated. The aquous phase was washed once with 20 mL of diethyl ether and the combined organic phases were dried over $MgSO_4$ and the solvent removed under vaccum. 0.257 mg of compound **2** (27% yield) were obtained by flash colum chromatography (cyclohexane to cyclohexane:EtOAc 19:1) as a slightly yellow liquid. Spectral data of compound **2** was in agreement with literature.[10] ^{1}H NMR (600 MHz, $CDCl_3$) δ=7.79 (m, 1H), 6.92 (dq, *J* = 4.0, 1.0 Hz, 1H), 2.61 ppm (s, 3H); ^{19}F NMR (565 MHz, $CDCl_3$) δ=-72.06 ppm.

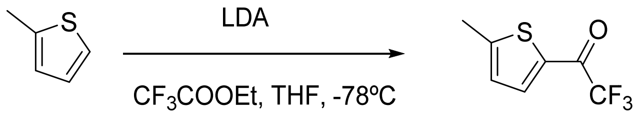

4 **2**

Scheme S3. Synthesis of monotrifluoromethyl ketone **2**.

***Procedure of the Photoligation Reaction*:** A NMR tube was loaded with 20 µL of a 5 mM solution of **2** in degassed toluene-d_8, 100 µL of a 30 mM solution of ***o*-MBA1** in degassed toluene-d_8 and 480 µL of degassed toluene-d_8. The NMR tube was irradiated for 1.5 hours (LED λ_{max} = 365 nm, 0.017 mW cm^{-2}) and the resulting mixture was analyzed by ^{19}F NMR spectrum every 15 min.

6. Light-induced Modulation of the *oxo*-Diels-Alder Photoligation between DTE1 and *o*-MBA1

***Procedure of the photoligation reaction*:** A NMR tube was prepared with 20 µL of a degassed toluene-d_8 solution 5 mM of $\mathbf{DTE1_{open}}$ in 0.5 mL of previously degassed toluene-d_8. When needed, $\mathbf{DTE_{closed}}$ was prepared in situ by irradiation for 5 min with a (LED λ_{max} = 365 nm**,** 23 mW cm^{-2}). Then, 100 µL of a 30 mM solution of ***o*-MBA1** in degassed toluene-d_8 was added and the sample was irradiated at selected conditions (LED λ_{max} = 365 nm at 0.017 mW cm^{-2}; LED λ_{max} = 625 nm from 0 to 1300 mW cm^{-2}). The progress of the photoligation reaction was monitored in time by ^{19}F NMR spectroscopy.

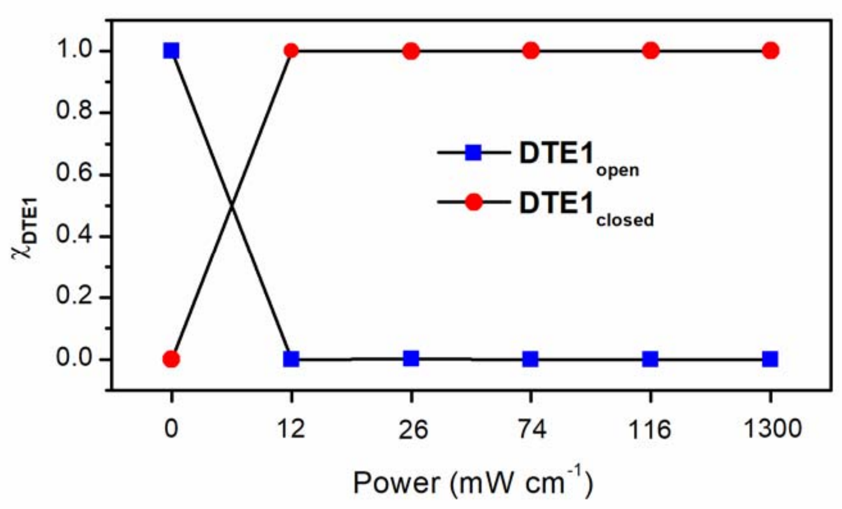

Figure S9. Variation of the composition of the photostationary state of **DTE1** in toluene (c = 0.16 mM) when simultaneously irradiated with UV (LED λ_{max} = 365 nm, 0.017 mW cm^{-2}) and visible light (LED λ_{max} = 625 nm, variable power: 0, 12, 26, 74, 116 and 1300 mW cm^{-2}).

7. NMR Spectra

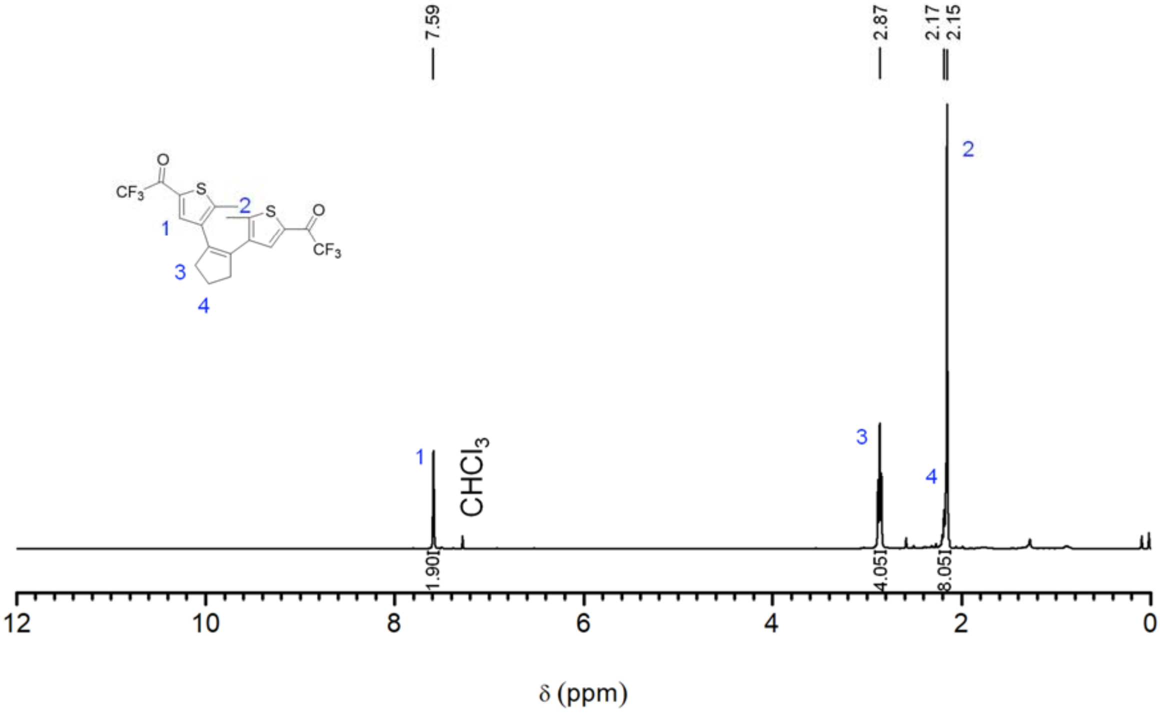

Figure S10. ^{1}H NMR spectrum (400 MHz, $CDCl_3$) of **DTE1$_{open}$**.

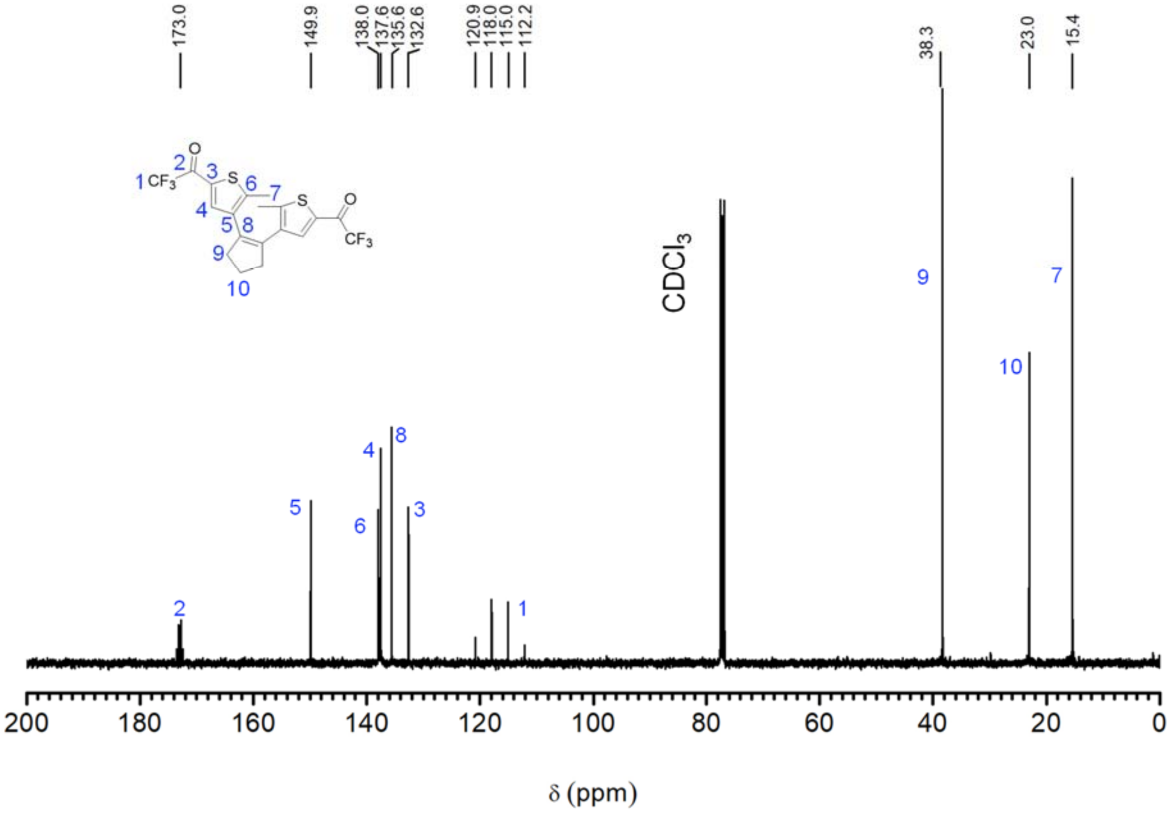

Figure S11. ^{13}C NMR spectrum (101 MHz, $CDCl_3$) of **DTE1$_{open}$**.

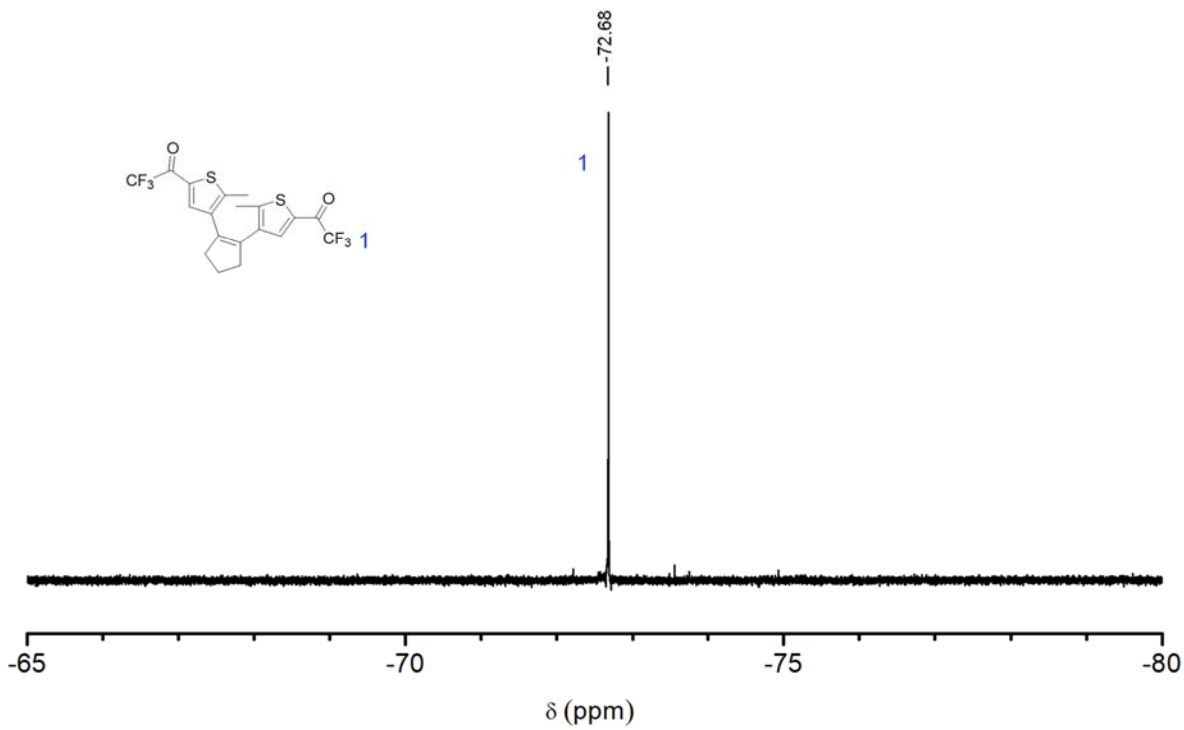

Figure S12. ^{19}F NMR spectrum (376 MHz, $CDCl_3$) of **DTE1$_{\mathbf{open}}$**.

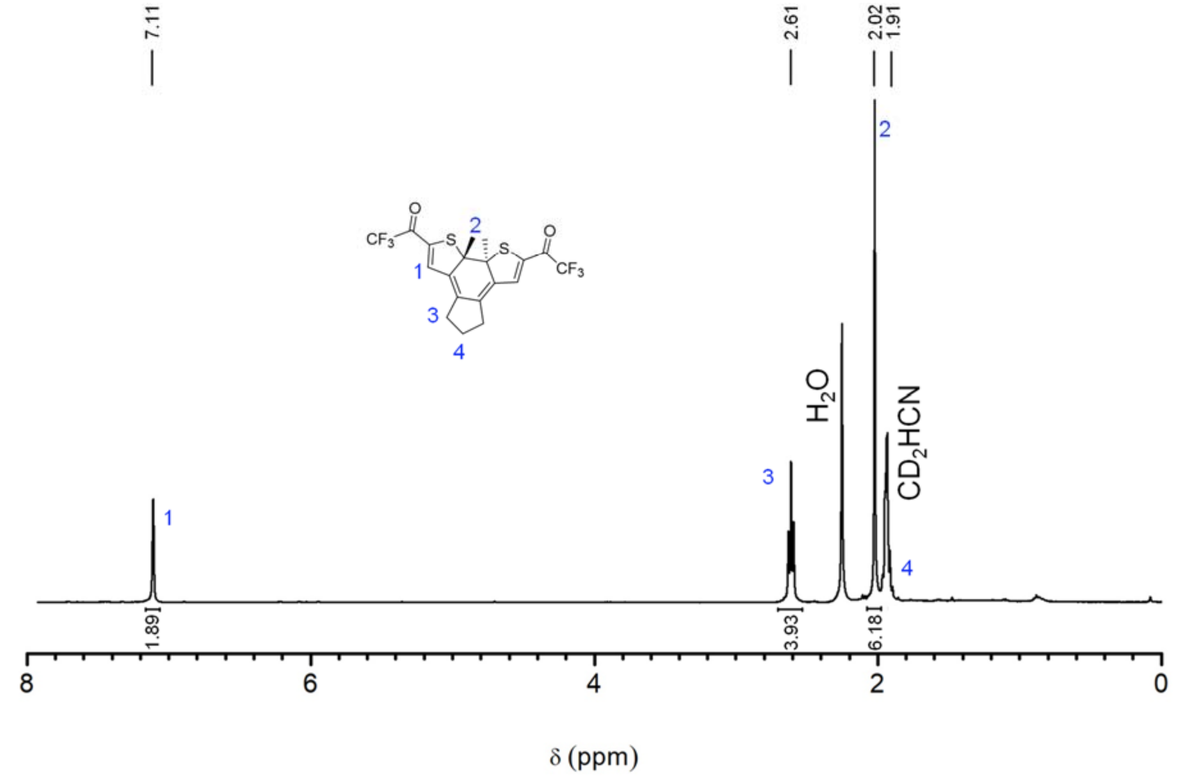

Figure S13. ^{1}H NMR spectrum (250 MHz, CD_3CN) of **DTE1$_{\mathbf{closed}}$**.

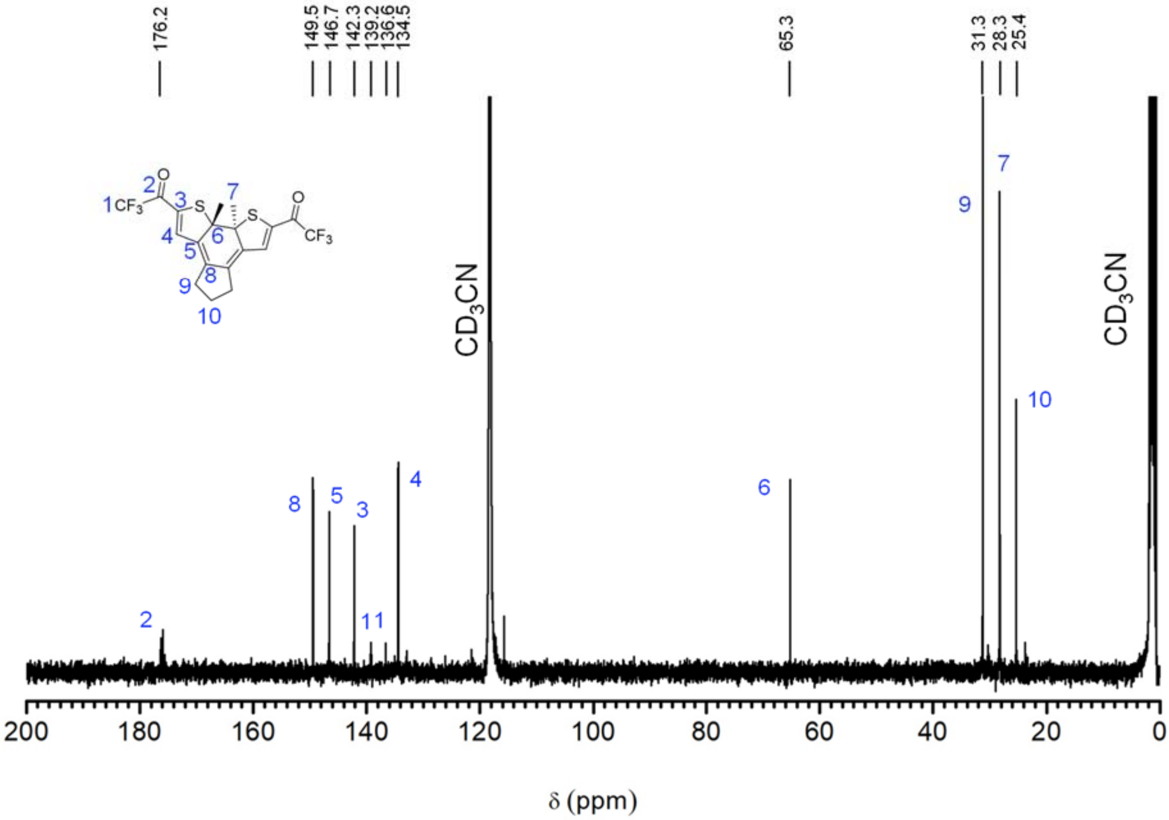

Figure S14. ^{13}C NMR spectrum (101 MHz, CD_3CN) of **DTE1$_{closed}$**.

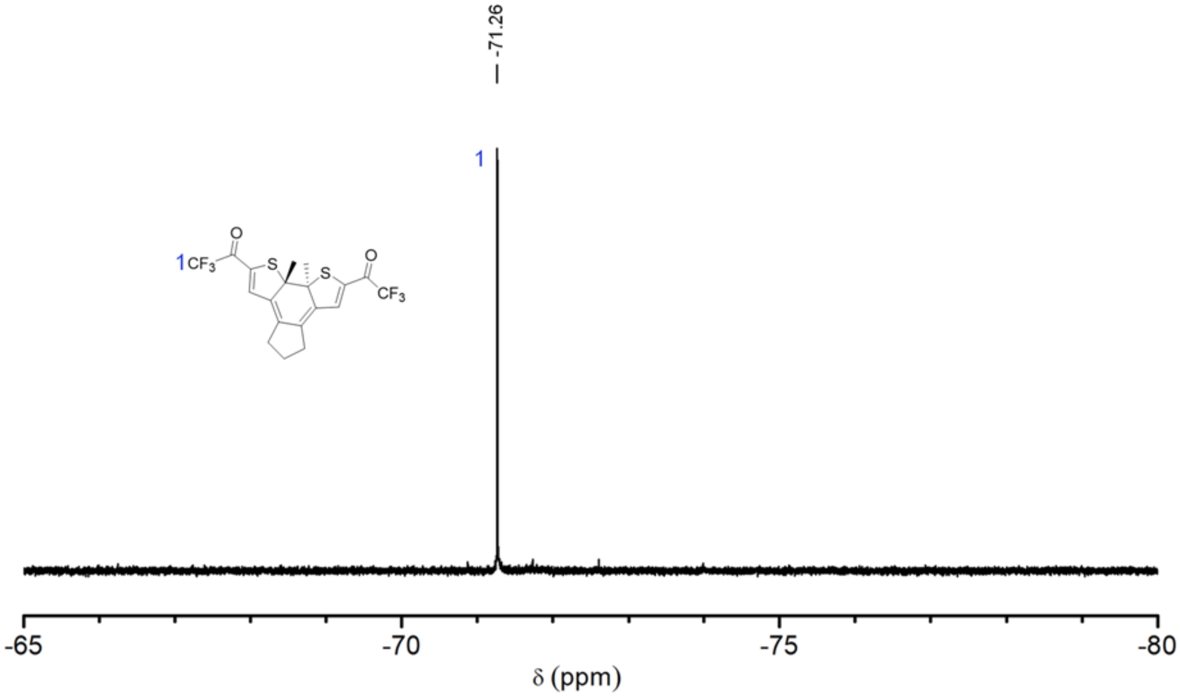

Figure S15. ^{19}F NMR spectrum (235 MHz, CD_3CN) of **DTE1$_{closed}$**.

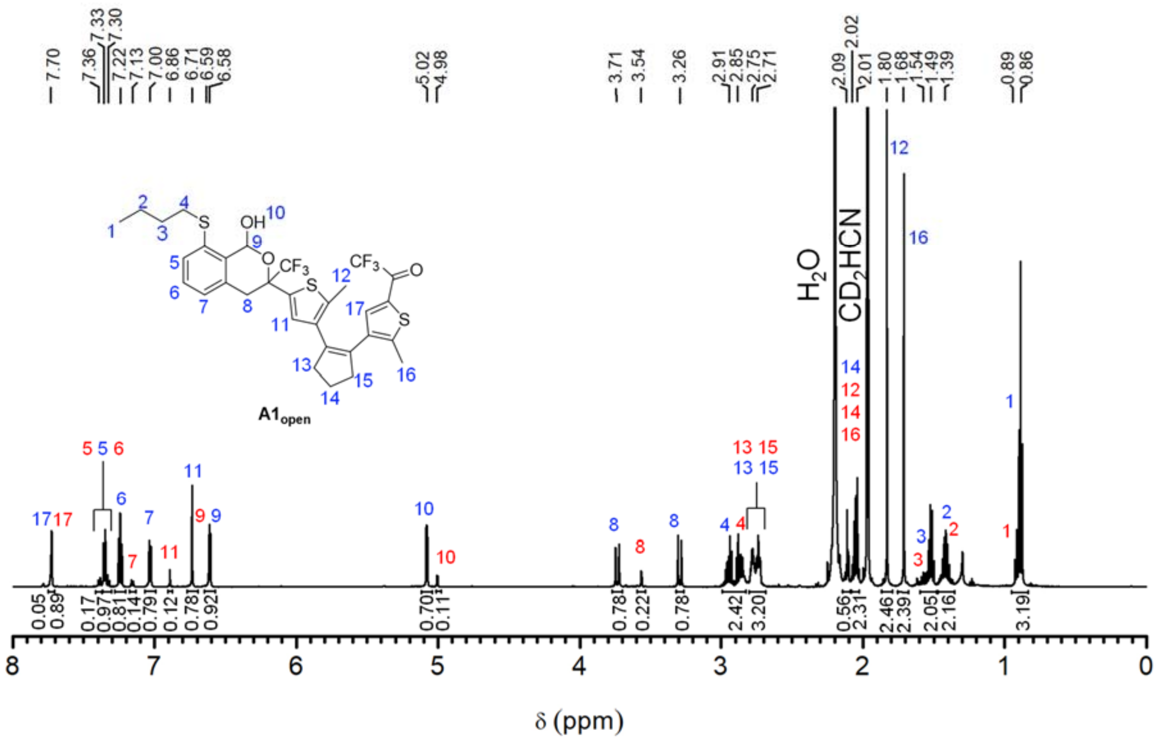

Figure S16 ^{1}H NMR spectrum (600 MHz, CD_3CN) of **A1$_{open}$**. Resonances are assigned in blue for **A1a$_{open}$** and red for **A1b$_{open}$**.

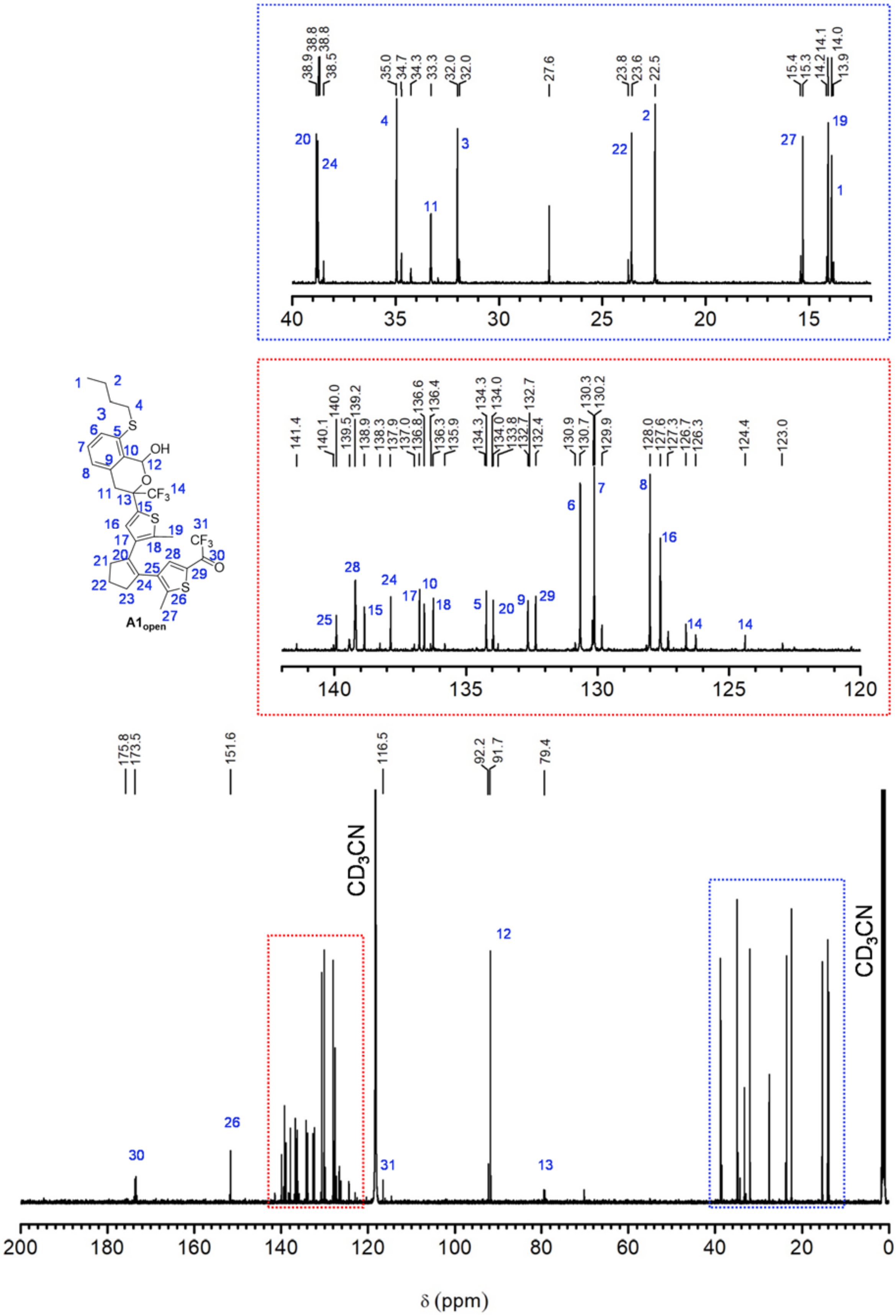

Figure S17. ^{13}C NMR spectrum (151 MHz, CD_3CN) of **A1$_{open}$**. Only the resonances for the major stereoisomer **A1a$_{open}$** could be assigned.

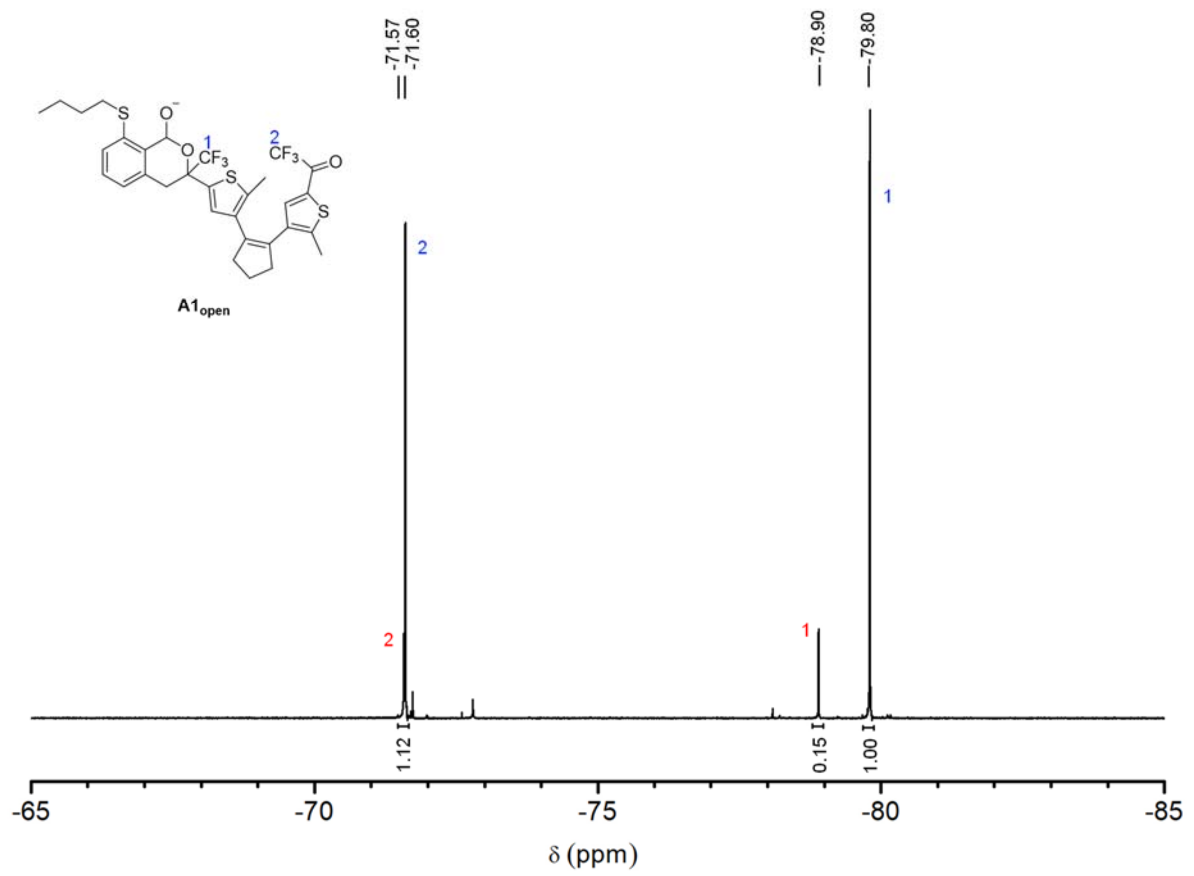

Figure S18. ^{19}F NMR spectrum (565 MHz, CD_3CN) of **A1$_{\mathbf{open}}$**. Resonances are assigned in blue for **A1a$_{\mathbf{open}}$** and red for **A1b$_{\mathbf{open}}$**.

8. References

(1) Sánchez, R. S.; Gras-Charles, R.; Bourdelande, J. L.; Guirado, G.; Hernando, J. Light- and Redox-Controlled Fluorescent Switch Based on a Perylenediimide–Dithienylethene Dyad. *J. Phys. Chem. C* **2012**, *116*, 7164–7172.

(2) Lees, A. J. A Photochemical Procedure for Determining Reaction Quantum Efficiencies in Systems with Multicomponent Inner Filter Absorbances. *Anal. Chem.* **1996**, *68*, 226–229.

(3) Higashiguchi, K.; Matsuda, K.; Asano, Y.; Murakami, A.; Nakamura, S.; Irie, M. Photochromism of Dithienylethenes Containing Fluorinated Thiophene Rings. *European J. Org. Chem.* **2005**, 91–97.

(4) Feist, F.; Rodrigues, L. L.; Walden, S. L.; Krappitz, T. W.; Dargaville, T. R.; Weil, T.; Goldmann, A. S.; Blinco, J. P.; Barner-Kowollik, C. Light-Induced Ligation of o - Quinodimethanes with Gated Fluorescence Self-Reporting. *J. Am. Chem. Soc.* **2020**, *142*, 7744–7748.

(5) Irie, M.; Fukaminato, T.; Matsuda, K.; Kobatake, S. Photochromism of Diarylethene Molecules and Crystals: Memories, Switches, and Actuators. *Chem. Rev.* **2014**, *114*, 12174–12277.

(6) Tietze, L. F.; Kettschau, G. Hetero Diels-Alder reactions in organic chemistry. In: P. Metz (ed) Stereoselective Heterocyclic Synthesis I. Topics in Current Chemistry, Springer, Berlin, **1997**, 189, 1–120.

(7) Jørgensen, K. A. Catalytic Asymmetric Hetero-Diels–Alder Reactions of Carbonyl Compounds and Imines. *Angew. Chem. Int. Ed.* **2000**, *39*, 3558–3588.

(8) Hentemann, M. F.; Allen, J. G.; Danishefsky, S. J. Thermal Intermolecular Hetero Diels–Alder Cycloadditions of Aldehydes and Imines Viao-Quinone Dimethides. *Angew. Chemie Int. Ed.* **2000**, *39*, 1937–1940.

(9) Takaki, K.; Fujii, T.; Yonemitsu, H.; Fujiwara, M.; Komeyama, K.; Yoshida, H. Hetero-

Diels–Alder Reaction of Photochemically Generated α-Hydroxy-o-Quinodimethanes with Trifluoromethyl Ketones. *Tetrahedron Lett.* **2012**, *53*, 3974–3976.

(10) Andicsová-Eckstein, A.; Kozma, E.; Puterová-Tokárová, Z.; Végh, D. Direct Trifluoroacetylation of Mono- and Disubstituted Thiophene Derivatives. *J. Fluor. Chem.* **2015**, *180*, 272–275.
